# Design and discovery of novel monastrol-1,3,5-triazines as potent anti-breast cancer agent *via* attenuating Epidermal Growth Factor Receptor tyrosine kinase

**DOI:** 10.1038/s41598-017-05934-5

**Published:** 2017-07-19

**Authors:** Jitendra Kumar Srivastava, Girinath G. Pillai, Hans Raj Bhat, Amita Verma, Udaya Pratap Singh

**Affiliations:** 1 Drug Design & Discovery Laboratory, Department of Pharmaceutical Sciences, Sam Higginbottom University of Agriculture, Technology & Sciences, Allahabad, Uttar Pradesh 211007 India; 2 Institute of Chemistry, University of Tartu, Ravila 14a, 50411 Estonia; 3 Florida Center for Heterocyclic Compounds, University of Florida, Gainesville, FL 32611 USA; 4 Department of Pharmaceutical Sciences, Dibrugarh University, Dibrugarh, Assam 786004 India

## Abstract

A novel series of hybrid analogues of monastrol-1,3,5-triazine were designed and developed *via* one-pot synthesis using Bi(NO_3_)_3_ as a catalyst. Entire compounds were evaluated for their anticancer activity against HeLa (cervical cancer), MCF-7 (breast cancer), HL-60 (Human promyelocytic leukemia), HepG2 (Hepatocellular carcinoma) and MCF 12A (normal epithelial breast cell line) using MTT assay, where they showed highest inhibitory activity against MCF-7. The molecules were also found to be non-toxic to MCF 12A cells. These molecules showed considerable inhibitory percentage against Epidermal Growth Factor Receptor tyrosine kinase (EGFR-TK), in *in-vitro* assay. Molecular docking study was carried out on the analogs and reference compound (Erlotinib) into the ATP binding site of EGFR-TK domain (PDB ID:1M17) to elucidate vital structural residues necessary for bioactivity. The effect of most active compound **7l** was also estimated *in-vivo* in DMBA induced mammary tumor in female Sprague-Dawley rats. The effect of anti-breast cancer effect of **7l** was quantified on the basis of tumour incidence, body weight and tumor volume in DMBA-induced rats. Its effect on biochemical parameters, such as antioxidant status (SOD, CAT, GPX and GSH) and lipid peroxidation was also studied. The compound **7l** showed inhibition of EGFR downstream signalling in the western blot analysis.

## Introduction

The search of novel medicinal agent endowed with therapeutic efficacy is always a great concern for the medicinal chemist. At the same time, the potential development of tolerance or resistance to that compound from the time it is first employed seriously compromised its clinical utility. This holds true and creates major hitch for the agents used in the treatment of chronic diseases such as cancer^1^.According to an estimate, Cancer is a second leading cause of morbidity and mortality after the cardiovascular diseases. It accounts for 12 million deaths across the globe by 2030, as per WHO^2^. Particularly in the developing countries, where resources are scarce, the impact of cancer on all populations is truly devastating. It became a serious concern for poor, vulnerable and socially disadvantaged people who get sicker and unable to afford expensive cancer medicines and treatments which die sooner as a result of it^3^. Modern day’s cancer research has been shifted towards the development of selective inhibitors which can able to target deregulated pathways to stop cancer growth in a classical drug–receptor fashion. This makes compounds less toxic to normal cells, and thus improves tolerability.

**Figure 1:**
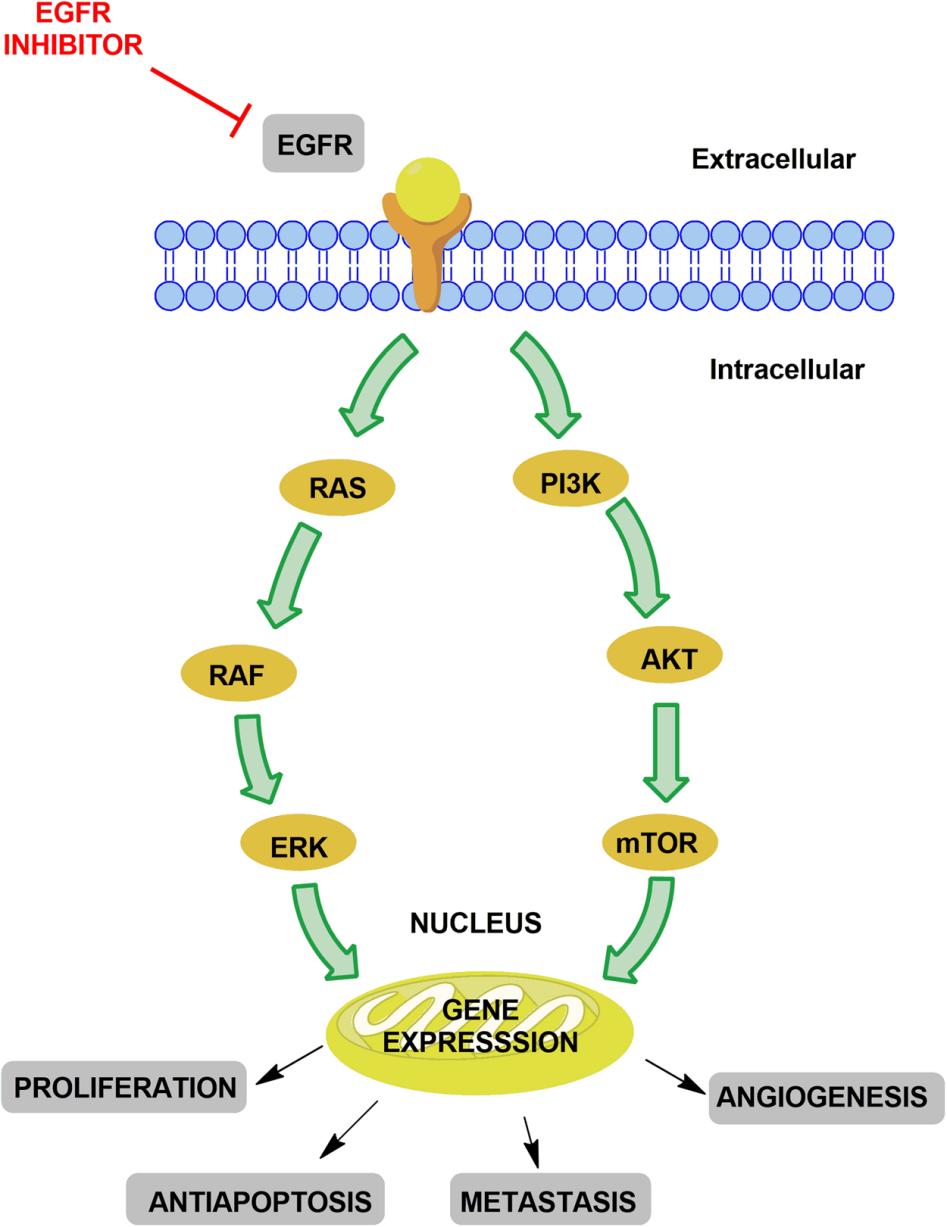
EGFR signalling pathway.

The epidermal growth factor receptor (EGFR) is a trans-membrane protein belonging to the erbB/HER-family of tyrosine kinase (TK) receptor, which includes four members defined as ErbB-1/EGFR/HER1, ErbB-2/HER2/neu, ErbB-3/HER3 and ErbB-4/HER4. These receptors share the same molecular structure with an extracellular, cysteine-rich ligand-binding domain, a single alpha-helix transmembrane domain, and an intracellular domain with TK activity in the carboxy-terminal tail (excepting the HER3)^4^. The receptors of EGFR family regulate the transcription of molecules that play a vital role in normal organ development by mediating morphogenesis and differentiation through effects on cell proliferation, differentiation, apoptosis, invasion, and angiogenesis. Unlike normal cells, the EGFR signalling has been aberrantly altered in tumour cells and often dysregulated. This behaviour leads to the proliferation of tumour cells under adverse conditions where they invade surrounding tissues, and thereby increases angiogenesis^5, 6^. EGFR intracellular signalling is mainly mediated by two interrelated downstream pathways, *viz*., the Ras-Raf-mitogen activated protein kinases (MAPK, also known as extra cytoplasmatic regulated kinases, ERK1 and ERK2) and the phosphatidylinositol 3-kinase (PI3K)/Akt pathways^7^. The overexpression of EGFR has been associated with advanced stages of many types of cancers especially breast, colon and bladder cancers^8^. Particularly in some breast cancer, subtypes The EGFR-dependent pathway appears to be a driver mechanism for malignant carcinogenesis, Fig. 1 ^9–11^. Thus, selective inhibition of this target by EGFR inhibitors offers various advantages.

**Figure 2:**
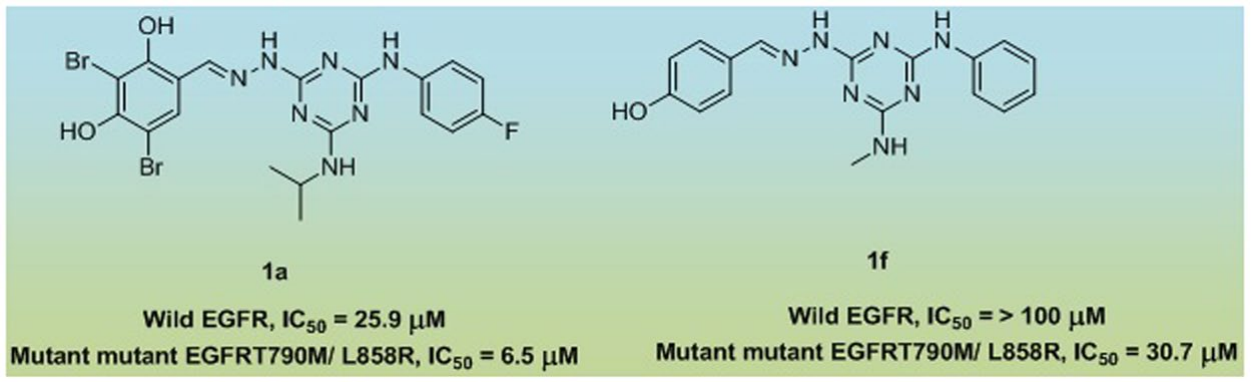
Some 1,3,5-triazine derivatives as Dual EGFR TK Inhibitor.

On the basis of hierarchical based virtual screening protocol, Bai *et al*., has identified number of 1,3,5-triazine derivative as dual-effective inhibitor against both WT and mutant EGFR TKs^12^. Out of them, compound **1a** exhibited most potent activity against WT EGFR (IC_50_ = 25.9 µM) and mutant EGFRT790M/L858R (IC_50_ = 6.5 µM), Fig. 2. Moreover, it also exhibit considerable antiproliferative activity against A549, A431 and NCIH1975 cell lines, IC_50_ = 7.7 ± 1.3, 8.0 ± 1.6 and 10.5 ± 0.9 µM, respectively. It has been found that that, when the fluoro group in the phenyl part was removed along with inclusion of the *para*-hydroxy group on the another phenyl, the inhibitory potency for WT EGFR has lost and reduced about fivefold for mutant EGFR, compound **1f**, Fig. 2. With the help of molecular docking analysis, they have shown that, the selectivity of molecules for WT EGFR and mutant EGFRT790M/L858R would be achieved by exploiting the additional hydrophobic pocket located in the back of the ATP-binding site by inclusion of other groups. This observation was found in agreement with the previous studies carried out by other researches on selective inhibitors against kinases^13, 14^.

**Figure 3:**
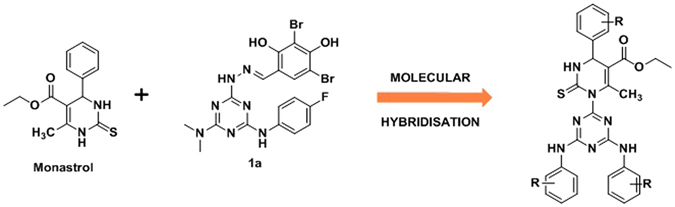
Design of target hybrid conjugates via molecular hybridisation.

Concerning our endeavour on discovery of novel chemotherapeutic agents from 1,3,5-triazine^15–22^ and its significance as EGFR TKs inhibitor, present study deals with advancement of novel 1,3,5-triazine derivatives as anticancer agents. The designed targeted molecules were developed *via* molecular hybridization of 1,3,5-triazine and Monastrol (3,4-dihydro-1*H*-pyrimidine) in a search of new hit. The later has been selected to get substituted on 1,3,5-triazine because of its anticancer potential and bulky nature, which could be tolerated at the active site of EGFR-TKs due its extended large hydrophobic cavity, Fig. 3. The synthesis of target hybrid compounds were accomplished by the means of one-pot synthesis using Bi(NO_3_)_3_ as catalyst.Consequently, the novel target molecules were also tested for anticancer activity.

## Results and Discussion

### Chemistry

To access heterocyclic compounds in fast and economic way along with high molecular diversity, one-pot synthetic approach has gaining attention. It is a synthetic strategy, whereby reactant/s is subjected to successive chemical reaction in a single reactor. This will improve the chemical reaction without isolating intermediates^10, 23–26^. Thus, considering the utility of one-pot reactions, the synthesis of the target compounds has been accomplished by utilizing Bismuth Nitrate as a catalyst.

**Figure 4:**
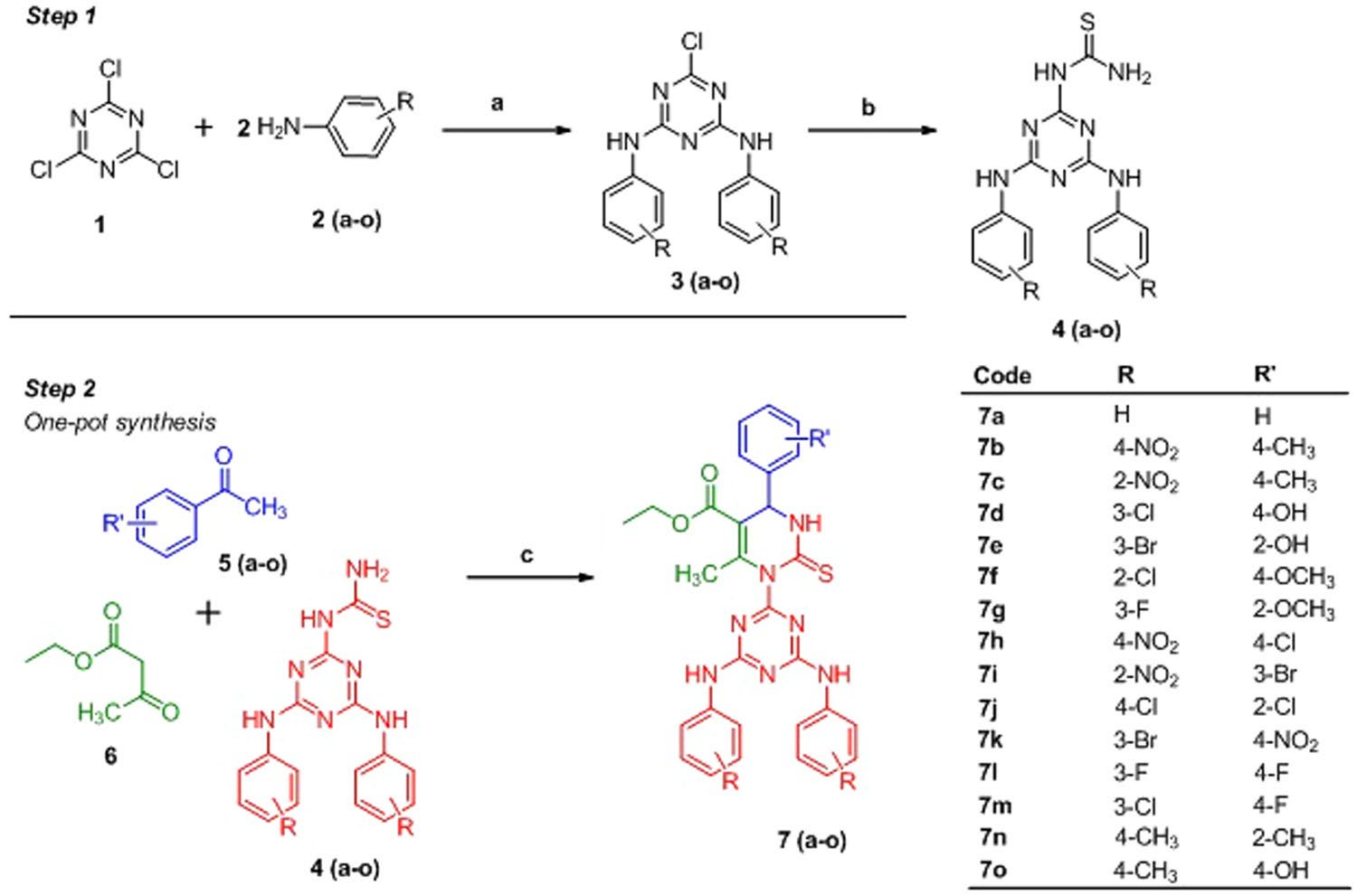
Reagents and condition, Step 1: Synthesis of 1-(4,6-bis(substituted phenylamino)-1,3,5-triazin-2-yl)thiourea derivatives **4** (**a–o**) a) NaOH, 40–45 °C, b) Reflux, 120–135 °C, K_2_CO_3_; Step 2: Biginelli’s one-pot condensation reaction **7**(**a–o**) c) Bi(NO_3_)_3_, reflux, ethanol.

As depicted in Step 1 (Fig. 4), the synthesis of 1-(4,6-bis(substituted phenylamino)-1,3,5-triazin-2-yl)thiourea derivatives **4** (**a–o**) were accomplished by reacting di-substituted 1,3,5-traizines **3** (**a–o**) with thiourea in the presence of the activating base at vigorous condition. While, compounds **3** (**a–o**) were synthesized *via* reacting cyanuric chloride (**1**) with two equivalents of substituted amines **2** (**a–o**) taking care that reaction mixture does not become acidic, with frequent addition of NaOH.

The next part of the study was aimed at optimising the reaction condition for the Biginelli’s one-pot condensation, Step 2, Fig. 4. In first instance, we tried to optimise the type of catalyst, where, we had taken numerous Lewis acids *e*.*g*. AlCl_3_, FeCl_3_, and ZnCl_2_ along with various bismuth salts *viz*., bismuth chloride, bismuth triflate, bismuth subnitrate, bismuth bromide, bismuth iodide and bismuth nitrate using benzaldehyde, ethyl acetoacetate (6) and 1-(4,6-bis(phenylamino)-1,3,5-triazin-2-yl)thiourea as a model reaction. The results were shown in Table 1.

**Table 1:** Optimisation of catalyst^a^.

Entry	Catalyst (10 mol%)	Time (h)	Yeild^b^
1	AlCl_3_	7	61
2	FeCl_3_	9	58
3	ZnCl_2_	8	60
4	BiCl_3_	6	67
5	Bi(OTf)_3_	5.30	84
6	BiI_3_	5.30	80
7	Bi_5_O(OH)_9_(NO_3_)_3_	5	89
8	BiBr_3_	5	75
9	Bi(NO_3_)_3_	4	92
10	No catalyst	16	34

^a^Reaction conditions: aldehyde (1 mmol), ethyl acetoacetate (1 mmol), 1-(4,6-bis(phenylamino)-1,3,5-triazin-2-yl)thiourea (2 mmol); solvent Ethanol; Reflux. ^b^Isolated and unoptimised yields.

During the process of optimising the catalyst, we found that 10 mol% of Bi(NO_3_)_3_ could effectively catalyse the reaction for the synthesis of desired product with maximum yield (92%) accompanied with shorter reaction time (4 h). The inclusion of other Bismuth salts *e*.*g*., BiBr_3_, Bi_5_O(OH)_9_(NO_3_)_3_, BiI_3_, Bi(OTf)_3_ and BiCl_3_ as a catalyst were not proved to be efficient enough and the reaction took longer time. It is remarkable to note that, the presence of various Lewis acids could not catalyses the reaction in an efficient way, *e*.*g*., FeCl_3_ take maximum of 9 h with 58% yield. While, in the absence of catalyst, the reaction did not precede smoothly, entry 10. This confirms the utility of the catalyst for the completion of reaction.

To divulge the role of solvent on the reaction and product yield, the next part of the study was aimed to determine the best solvent. Consequently, the reaction has been carried out with different solvents and the results were shown in Table 2. It was inferred that ethanol furnish the product in highest yield with shorter reaction time, entry 5. The time taken to complete the reaction has been augmented two-fold in the case of THF (entry 2) and toluene (entry 3) in comparison to ethanol and further increased in the case of dichloromethane (entry 4). While, the water was proved as ineffective solvent to this type of transformation owing to its high reaction time and less product yield, entry 1.

**Table 2:** Optimisation of the solvent^a^.

Entry	Solvent	Yield^b^	Time
1	Water	52	11
2	THF	70	8
3	Toluene	73	8
4	Dichloromethane	69	10
5	Ethanol	92	4

^a^Reaction conditions: aldehyde (1 mmol), ethyl acetoacetate (1 mmol), 1-(4,6-bis(phenylamino)-1,3,5-triazin-2-yl)thiourea (2 mmol); solvent Ethanol; Reflux. ^b^Isolated and unoptimised yields.

After optimisation of the catalyst (Bi(NO_3_)_3_) and solvent (ethanol), numerous target products containing diverse structural motifs has been synthesized as shown in Fig. 4. The synthetic procedure came out as very straight forward; the target products were isolated and purified by simple filtration and column chromatography.

### Anticancer activity

The synthesized compounds were evaluated for *in-vitro* cytotoxic activity against various cell lines such as HeLa (cervical cancer), MCF-7 (breast cancer), HL-60 (Human promyelocytic leukemia), HepG2 (Hepatocellular carcinoma) and MCF 12A (normal epithelial cell) by the MTT assay method. Cisplatin, one of the most effective anticancer agents was used as a reference drug in this study. The relationship between surviving fraction and drug concentration was plotted to obtain the survival curve of all the cancer cell lines HeLa, MCF-7, HL-60 and HepG2. The response parameter calculated was the IC_50_ values, which responds to the concentration required for 50% inhibition of cell viability. The *in-vitro* cytotoxic activity of the synthesized compounds is summarized Table 3.

**Table 3:** Anticancer activity of target compound.

IC_50_ (in µM)					
Compound	HeLa	MCF-7	HL-60	HepG2	MCF 12A
7a	96.6 ± 0.45	87.4 ± 0.76	83.7 ± 0.27	NA	non-toxic
7b	81.8 ± 0.34	76.5 ± 0.54	77.3 ± 0.41	79.3 ± 0.75	non-toxic
7c	83.5 ± 0.22	72.2 ± 0.61	70.5 ± 0.49	86.4 ± 0.67	non-toxic
7d	51.3 ± 0.32	61.3 ± 0.78	43.2 ± 0.38	41.4 ± 0.38	non-toxic
7e	63.9 ± 0.25	67.8 ± 0.61	57.4 ± 0.52	65.6 ± 0.45	89.45 ± 0.45
7 f	55.4 ± 0.12	64.0 ± 0.38	47.0 ± 0.54	46.5 ± 0.36	82.14 ± 0.21
7 g	42.1 ± 0.12	47.4 ± 0.27	31.5 ± 0.21	36.3 ± 0.47	77.22 ± 0.34
7 h	73.4 ± 0.65	71.5 ± 0.46	61.4 ± 0.36	69.6 ± 0.38	non-toxic
7i	76.0 ± 0.54	76.4 ± 0.33	66.7 ± 0.67	71.6 ± 0.29	non-toxic
7j	46.2 ± 0.56	57.0 ± 0.47	39.6 ± 0.78	38.7 ± 0.32	69.13 ± 0.62
7k	66.4 ± 0.67	65.6 ± 0.43	51.3 ± 0.82	59.7 ± 0.69	non-toxic
7l	39.7 ± 0.81	41.5 ± 0.31	23.1 ± 0.36	31.2 ± 0.82	non-toxic
7m	42.1 ± 0.34	53.8 ± 0.28	36.5 ± 0.48	39.3 ± 0.12	73.56 ± 0.40
7n	92.6 ± 0.23	93.2 ± 0.58	104.5 ± 0.39	NA	non-toxic
7o	94.3 ± 0.78	89.4 ± 0.49	98.3 ± 0.27	NA	non-toxic
Cisplatin	32.5 ± 0.62	24.4 ± 0.92	12.3 ± 0.76	25.9 ± 0.82	non-toxic

NA: Not active.

Results showed that absence of the substituent *i*.*e*., intact phenyl rings render the compound inactive against HepG2, while less active against rest of the cell lines, **7a**. In the case of compounds **7b** and **7c**, against entire cell lines, no significant change in activity has been observed. To our surprise, the activity was significantly increased against HepG2 on the introduction of 3-Cl at the phenyl of the triazine along with electron donating group (4-OH), compound **7d**. Whereas, on replacing chloro with bromo along with shifting of hydroxyl group to *meta*, the activity was dropped, **7e**. Minor upsurge in activity was reported in the case of compounds **7f** and **7g** aganist entire cell lines. Presence of non-halogen substituent at the phenyl of triazine along with halogen on the other counterpart significantly lowers the activity against all tested cell lines, compounds **7h** and **7i**. A momentous increase in activity was reported by the compounds **7j**, **7l** and **7m** having halogen groups except mild activity in the case of compound **7k** having NO_2_ as one the substituent. It is noteworthy to mention that, presence of electron donating substituent significantly diminishes the anticancer potential and makes the compounds inactive against HepG2. All the tested hybrid conjugates exhibited moderate to significant anticancer activity except compound **7a**, **7n** and **7o** for HepG2. Results showed that none of the synthesized compounds exhibited pronounced activity than cisplatin as standard.

**Figure 5:**
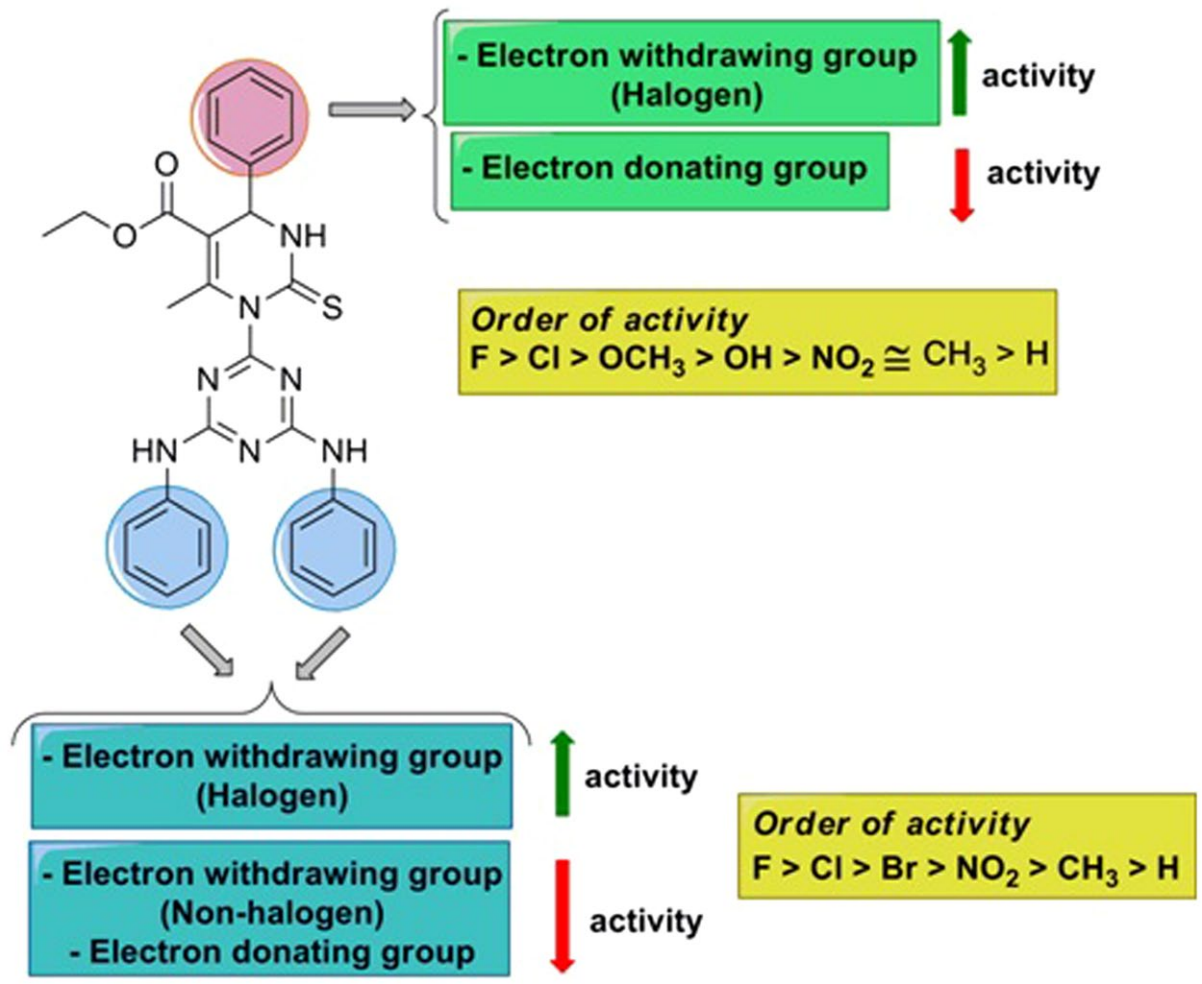
Structure-activity relationship study of **7** (**a–o**) as anticancer.

It was corroborated that, compound **7l** was emerged as most active molecule while compound **7n** and **7o** as non-active against the entire tested cell lines. Structure-activity relationship studies suggest that presence of halogen electron withdrawing group is necessary for generation and escalation of activity. The presence of non-halogen substituent along with electron donating substituent seriously jeopardise the anticancer potential. Results showed a clear pattern of the effect of substituent on the activity and the same has been discussed in Fig. 5. To determine the selectivity of these molecules between normal and cancerous cells, the designed compounds were also tested against MCF 12A, a normal epithelial breast cell. The results showed that, most of molecules found non-toxic to the normal cell.

### EGFR-TKs Inhibitory Activity

The synthesized 1,3,5-triazine-3,4-dihydro-1*H*-pyrimidine derivatives were evaluated *in vitro* for EGFR-TK enzyme inhibitory activity (at 10 µM) and the results obtained are reported as percent of inhibition in comparison to Erlotinib as a standard. The results were displayed in Table 4. Against EGFR-TKs, entire set of target compounds showed excellent inhibitory activity, where, compound **7l** and **7g** revealed as most potent inhibitor with 96.4% and 94.3%, respectively. Further decline in activity was reported by compound **7m** (88.5%) and **7f** (81.4%). A significant drop in inhibitory potency was reported by compound **7e** and **7j** which was followed by further reduction against EGFR-TK by **7e** and **7d** (78.5% and 74.8%, respectively). It was surprising to note that similar pattern of inhibition was disclosed by compound **7h**, **7i** and **7k** near to 69%. The assay revealed that, rest of compound showed inhibitory percentage nearly half to that of Erlotinib, except compound **7o** which showed least inhibition, i.e., 45.6%.

**Table 4:** EGFR tyrosine kinase inhibitory activity of target hybrid derivatives at 10 µM.

Compound	Percent of Inhibition
7a	52.4
7b	56.7
7c	53.6
7d	74.8
7e	78.5
**7 f**	**81**.**4**
**7 g**	**94**.**3**
7 h	69.1
7i	69.3
7j	82.4
7k	70.1
**7l**	**96**.**4**
**7m**	**88**.**5**
7n	54.2
7o	45.6
**Erlotinib**	**100**

### Molecular Docking Study

#### Binding Analysis of inhibitors docked to EGFR-TK domain

Captivated by the exceptional anticancer activity shown by target molecules against MCF-7 (breast cancer) where EGFR enzyme is over-expressed, and potent EGFR-TKs inhibitory activity, we used binding information of our receptor against EGFR-TK inhibitors for validation of our results. Present study was based on genetic algorithm and *in-silico* approaches. The details of ligand docking scores are given in Table 5.

**Table 5:** Docking results with energies of all ligands.

Properties/Ligand	B.E. (kcal/mol)	%i	pKi (µM)	L.E.	I.E. (kcal/mol)	vHd.E. (kcal/mol)	E.E. (kcal/mol)	TI.E./US.E. (kcal/mol)	TF.E. (kcal/mol)
**7a**	−8.56	52.4	528.02	−0.21	−11.25	−10.54	−0.71	−1.37	2.68
**7b**	−8.81	56.7	350.66	−0.18	−12.09	−10.54	−1.55	−1.89	3.28
**7c**	−8.30	53.6	828.73	−0.17	−11.88	−10.71	−1.16	−4.07	3.58
**7d**	−8.38	74.8	716.95	−0.19	−11.66	−11.97	0.3	−0.47	3.28
**7e**	−9.25	78.5	166.58	−0.21	−12.53	−11.21	−1.32	−0.54	3.28
**7 f**	−9.57	81.4	96.44	−0.21	−12.55	−11.84	−0.71	−0.44	2.98
**7 g**	−7.83	94.3	1.83	−0.17	−11.11	−10.20	−0.91	−1.28	3.28
**7 h**	−8.33	69.1	777.01	−0.17	−11.62	−10.30	−1.32	−0.46	3.28
**7i**	−9.28	69.3	158.23	−0.19	−12.56	−11.39	−1.17	−3.71	3.28
**7j**	−8.64	82.4	463.48	−0.20	−11.62	−10.55	−1.08	−1.04	2.98
**7k**	−8.74	70.1	394.82	−0.19	−12.02	−10.45	−1.56	−1.8	3.28
**7l**	−8.85	96.4	326.97	−0.2	−11.83	−10.68	−1.15	−1.41	2.98
**7m**	−9.50	88.5	109.61	−0.22	−12.18	−11.33	−0.85	−1.29	2.68
**7n**	−8.89	54.2	301.94	−0.2	−11.58	−10.68	−0.9	−1.91	2.68
**7o**	−8.79	45.6	362.71	−0.2	−12.07	−11.28	−0.79	−1.76	3.28
**ERL**	−7.47	100	3.37	−0.26	−10.45	−10.32	−0.13	−1.29	2.98

B.E. = Binding Energy, pKi = Predicted Inhibition Constant, I.E. = Intermolecular Energy, vHd.E. = vdW + Hbond + desolv Energy, E.E. = Electrostatic Energy, TI.E. = Final Total Internal Energy, US.E. = Unbound System’s Energy, TF.E. = Torsional Free Energy.

Docking study was performed to understand the interactions of the compounds with the active center of the EGFR-TK receptor by validating the binding mode and interacting amino acids from the active conformation of the co-crystal ligand Erlotinib. We selected PDB ID: 1M17 as our target protein and the protein had co-crystal ligand Erlotinib with literature reference on binding affinities and the protein receptor EGFR-TK was our focused target^27^. While comparing the docking results of ligands, we observed that erlotinib, **7l**, **7m**, **7e** has the lowest binding energy and predicted inhibition constant values as more negative binding energy score (kcal/mol) corresponds to the binding affinity. On the basis of lower binding free energy, better electrostatic interaction and inhibition constant value of these inhibitors against EGFR-TK, we came to know that **7l** and **7m** are inhibitors with efficacy for EGFR-TK inhibition. Also the interaction data supported this expectation as **7l** and **7m** showed maximum number of hydrogen bonds within the receptor binding site and along with hydrophobic regions as compared to other ligands (Table 5).

**Figure 6:**
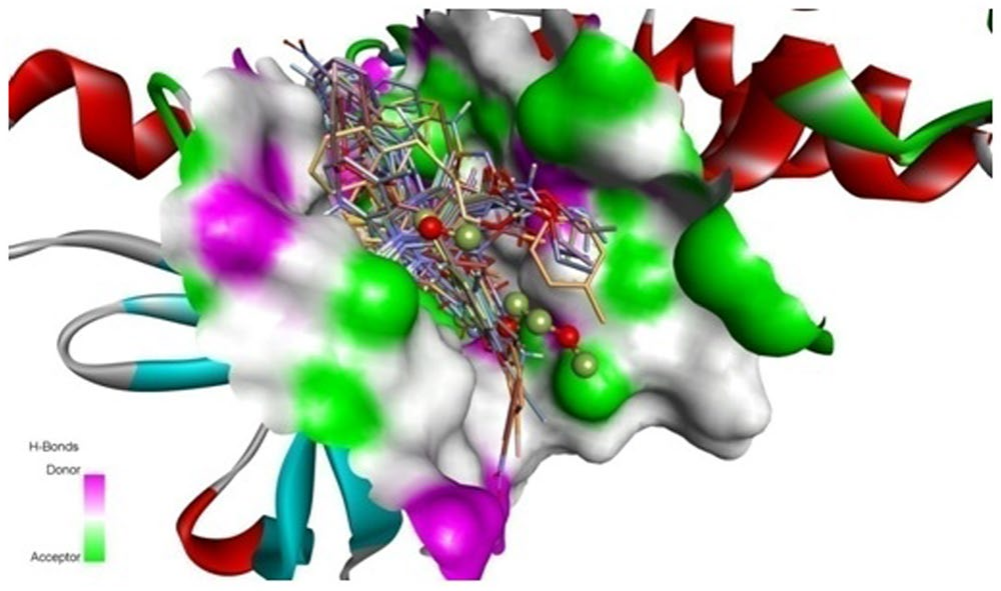
Protein EGFR-TK (PDB:1M17) complex with 7a-7o (stick) compounds including the ERL co-crystal ligand (stick and ball).

**Figure 7:**
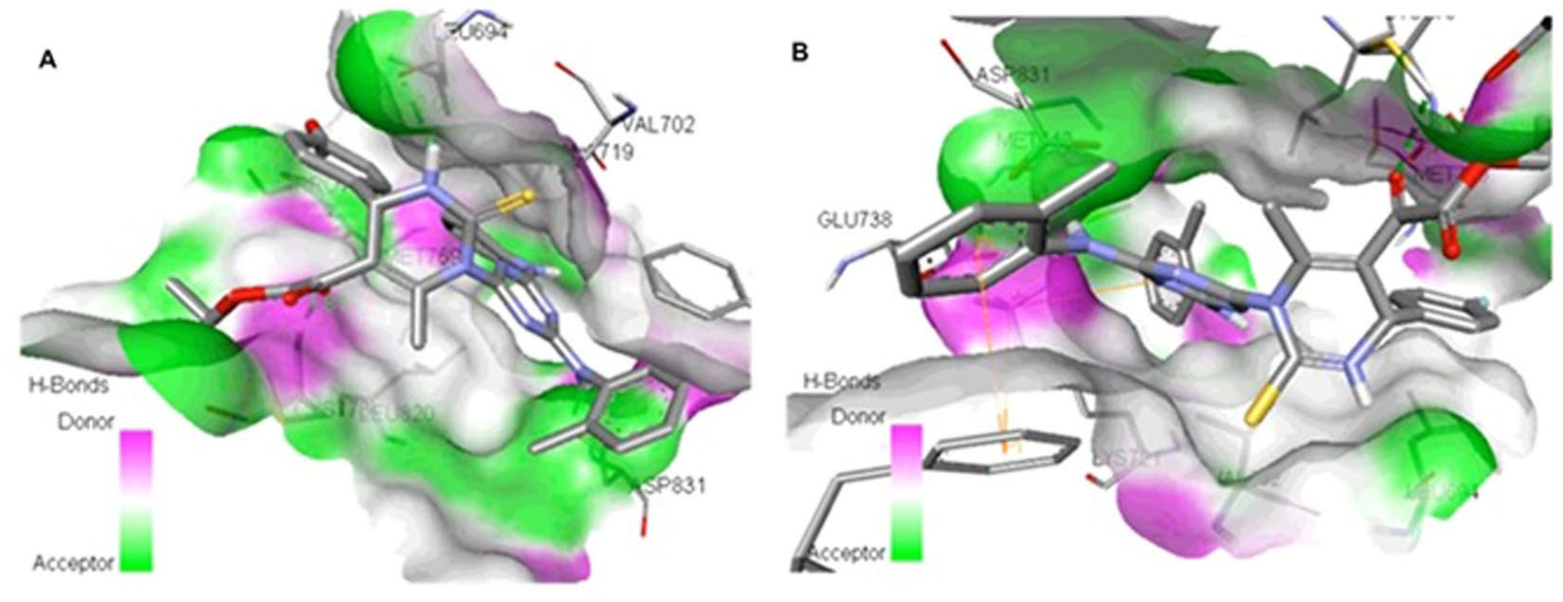
3D representation of the receptor-ligand interactions of 7f (**A**) and 7m (**B**) compounds. Interaction details of all compounds are given in Table S1 of SI.

The binding mode between EGFR tyrosine kinase domain target and the molecules **7a** to **7o** including ERL, the structures in complex derived from the best dock results are given in Fig. 6. The hydrogen bond donor, acceptor along with hydrophobic regions of the compounds **7a** to **7o** was found to be oriented within the active center of target protein binding site. The top ligands **7l**, **7m**, **7e**, and **7f**, evaluated by least binding affinity more than −8.82 Kcal/mol which indicated the effective binding. The moderate binding was observed in the case of remaining ligands with binding energy found to be in the range of −7 to −8.80 kcal/mol. From the docking, it was suggested that there are key H-bonds created via interacting with MET769, ASP831, LYS721 and CYS773 amino acids in the EGFR-TK - **7l**/**7m** complexes (Table S1(l,m)), while **7e** and **7f** (Table S1(e,f)) has H-bonds with THR766, GLN767, THR830 apart from the other key amino acid residues. The reference molecule Erlotinib (ERL) (Table S1(ERL)) have one key H-bond with MET769 AA. Among the binding residues, those involved in hydrogen bonds with the inhibitors are detailed with type of hydrogen bond formed (in context of hydrogen bond donor and acceptor) and distance between the atoms forming them. 3D representation of **7f** and **7m** compounds are given in Fig. 7. A 2D depiction of the interactions of binding residues of receptor protein with the ligands and H-bond distances are represented in Table S1 of Supporting Information. Results indicated that **7f**, **7l** and **7m** intensively bind EGFR-TK through H-bonds and hydrophobic interactions. Molecules including **7l**, **7m**, **7f**, **7g** had longer and broader structures when compared with the co-crystal ligand ERL and the protein binding pocket size. This was considerable reason for slight discrepancies in the binding energies while comparing with experimental data. Therefore compounds like **7l** and **7g** showed significantly decreased binding energies and favourable inhibition. Docking simulation was performed to provide a molecular level foundation to illustrate and position compounds into the EGFR active site to determine the probable binding conformation at the active site of EGFR tyrosine kinase.

### In *vivo* pharmacology

**Figure 8:**
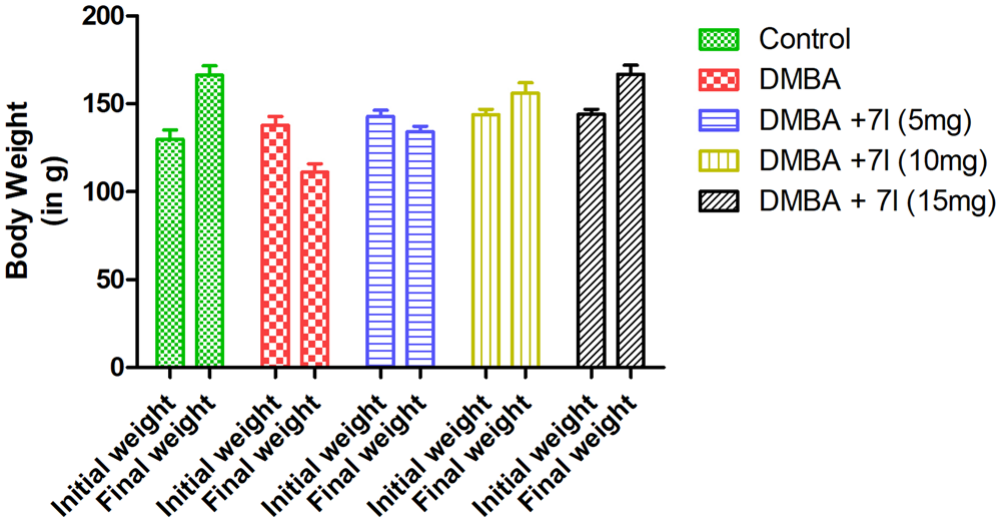
Effect of compound 7l on the body weight of control and treated animal groups.

Encouraged by the excellent EGFR-TK inhibitory activity and anticancer effect of compound **7l** against breast cancer cells which are further substantiated with docking analysis, prompted us to further analyze the effect of compound **7l** in the *in-vivo* system. Therefore, we have utilized 7,12-Dimethylbenz(a)anthracene (DMBA), a polycyclic aromatic hydrocarbon, to induce the mammary carcinogenesis in the experimental animals to quantify the potential protective effect of compound **7l**. The mechanism behind its action to induce the cancer is based on its metabolized product, i.e. the epoxide that readily forms DNA adducts which is responsible for malignant alteration. At the first instance, the effect of compound **7l** was analyzed on the effect of body weight of the experimental animals. As shown in Fig. 8, the body weight of the DMBA treated group showed considerable decline in the weight as compared to the control due to reduction in the normal metabolic process needed for maintaining the energy balance. Moreover, the body weight of the experimental animals has been significantly improved in the case of **7l** treated group in a dose-dependent manner.

**Figure 9:**
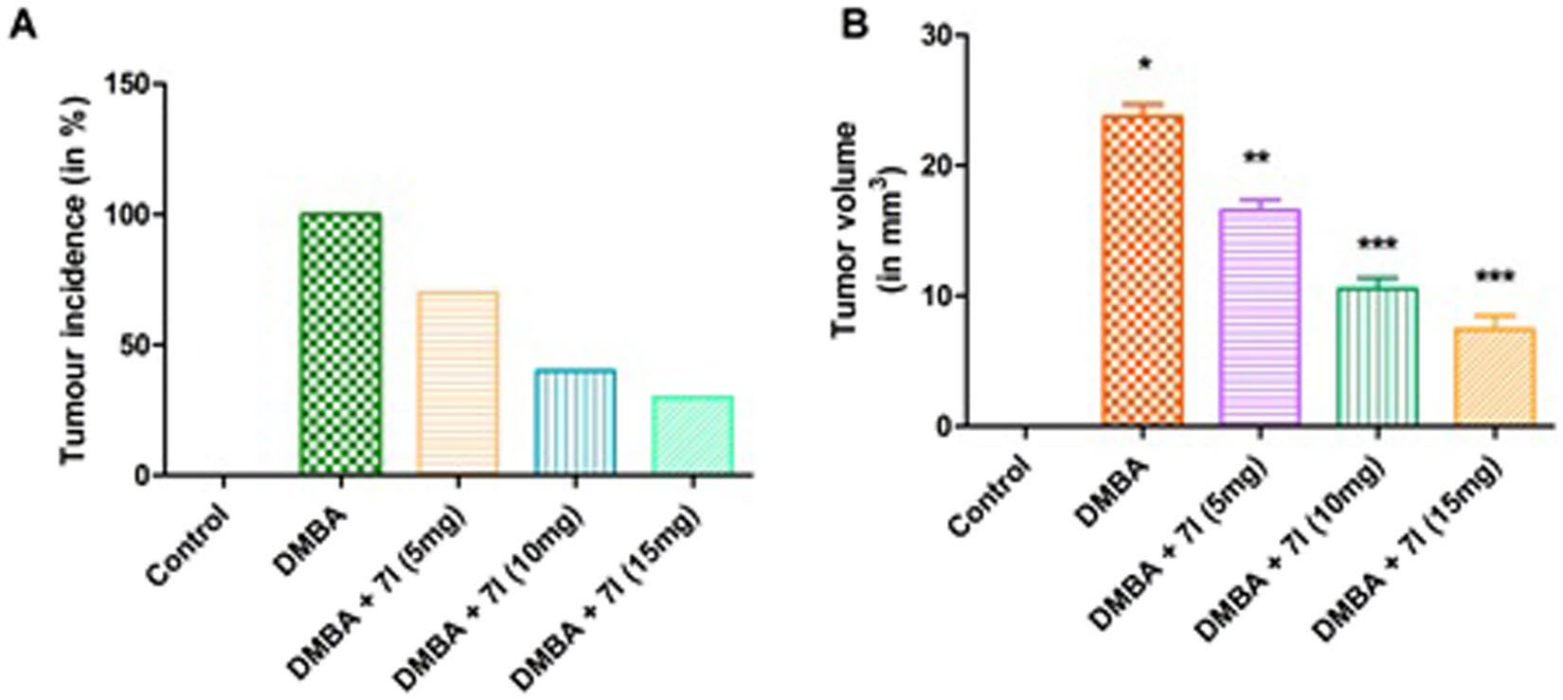
Effect of compound 7l on the tumor incidence (**A**) and tumour volume in different animal groups. *P < 0.05 is considered as significant, **P < 0.01 is considered as very significant, ***P < 0.001 is considered as extremely significant.

The effect of compound **7l** was also investigated on the percentage of tumour incidence and on the tumour volume. The results of the study was clearly elucidated in the Fig. 9, which indicated that, compound **7l** was efficient in reducing the percentage of incidence of tumour in cancer bearing animals. Moreover, **7l** was also showed significant reduction in the tumour volume of the cancer induced experimental groups. The results suggests clearly suggest the broad protective effect of compound **7l** against the mammary cancer.

**Figure 10:**
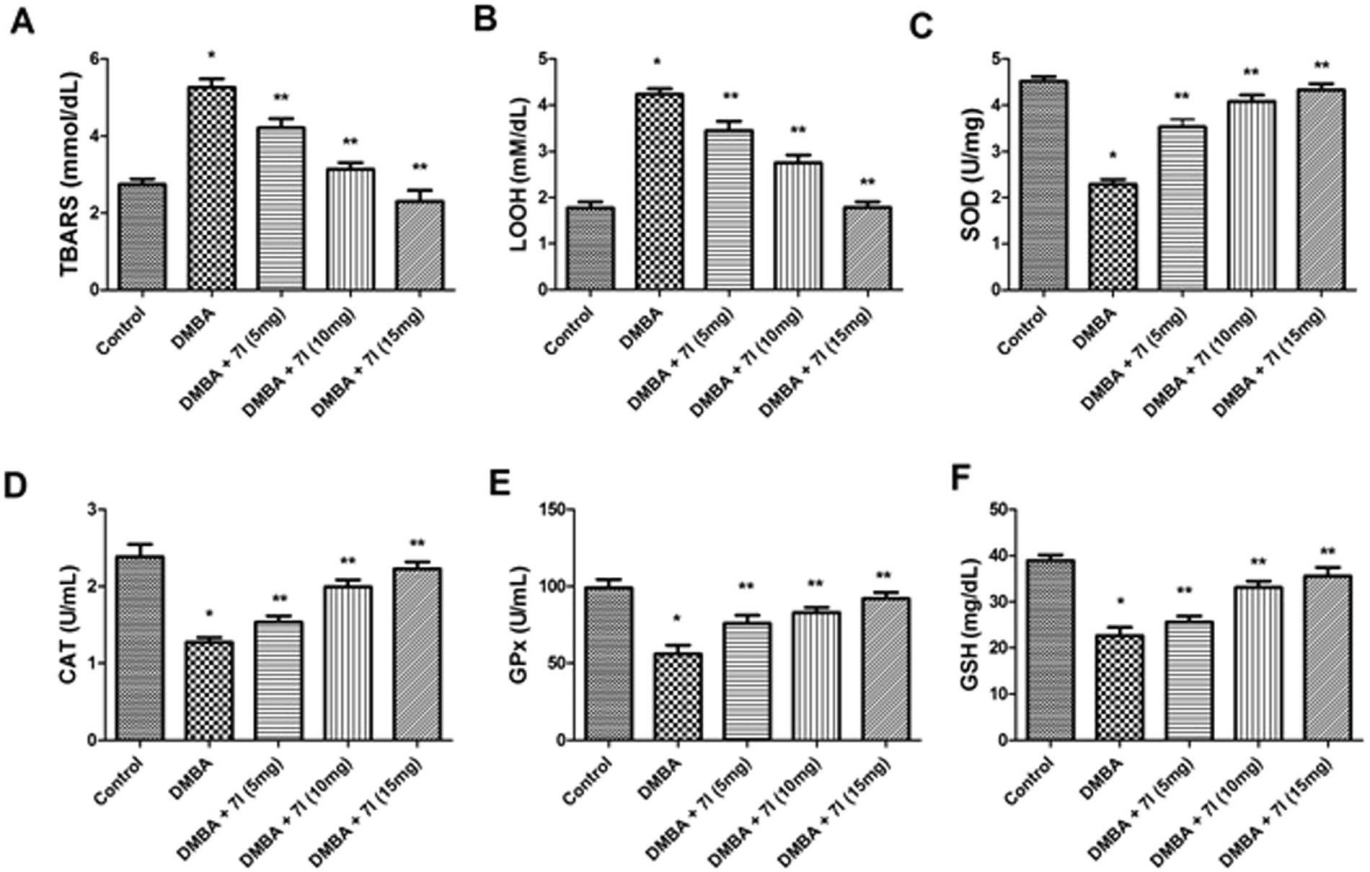
Effect of compound 7l on the antioxidant status in the plasma of treated animals and control. *P < 0.05 is considered as significant, **P < 0.01 is considered as very significant.

**Figure 11:**
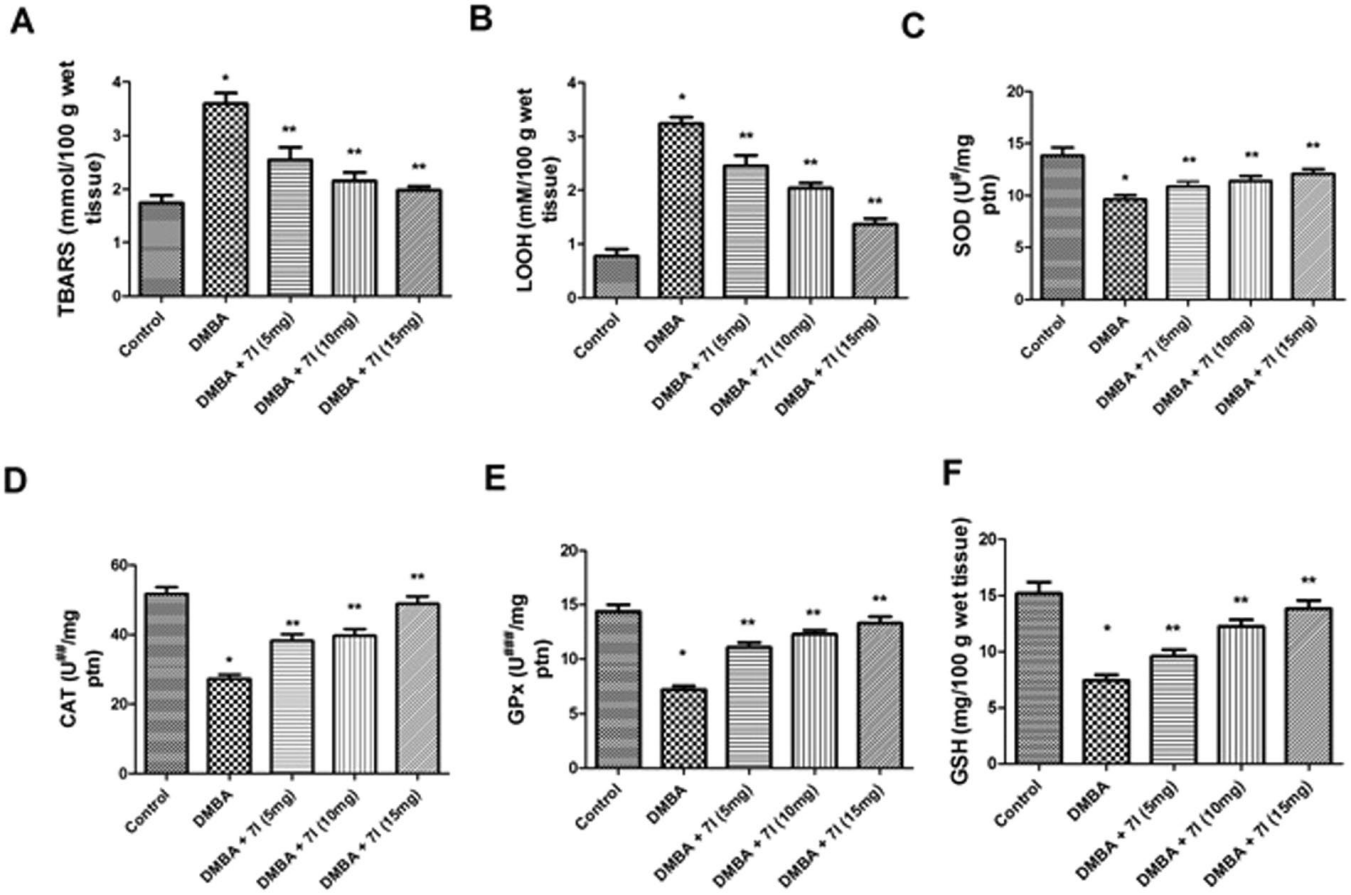
Effect of compound 7l on the antioxidant status in the mammary tissue of treated animals and control. *P < 0.05 is considered as significant, **P < 0.01 is considered as very significant.

The next part of the study was aimed at elucidating the effect of the compound **7l** on the antioxidant status of the experimental animals. Previous studies have confirmed the role of antioxidant system as a primary line of defence against cancer inducing agent. Therefore, this study has vital implication to understand the role of **7l** against the breast cancer because; the effectiveness of endogenous anti-oxidant system has been greatly compromised and often aberrantly deregulated. In the present study, we observe a significant decline in the level of endogenous anti-oxidative enzymes, such as, SOD, CAT, GSH and GPx. As shown in fig 10 and 11 in plasma and mammary tissues, respectively, the level of these enzymes in DMBA alone treated group was significantly reduced as compared to the control. Whereas, on admistration of compound **7l** showed significant improvement in the antioxidant status of the DMBA treated animals. However, when these results are correlated with tumour incidence and volume, it has been suggested, the compound **7l** showed considerable protection against the tumour and exert its protective action against breast cancer probable *via* scavenging the free radical. The lipid peroxidation has been greatly influenced by the generation of reactive oxygen species (ROS) and have profound role in the progression of the tumour. Therefore, in the next instance, the effect of compound **7l** was determined on the level of TBARS and LOOH, which considered as critical biomarker for the assessment of peroxidation of membrane lipids. As evident from the Figs 10(A and B) and 11(A & B), it was clear that, the level of TBARS and LOOH has been found to be significantly elevated as compared to control. Moreover, it was marked to note that, the administration of **7l** causes significant reduction in the level of these investigated markers, which further found to be suitably correlated with improvement in antioxidant system.

**Figure 12:**
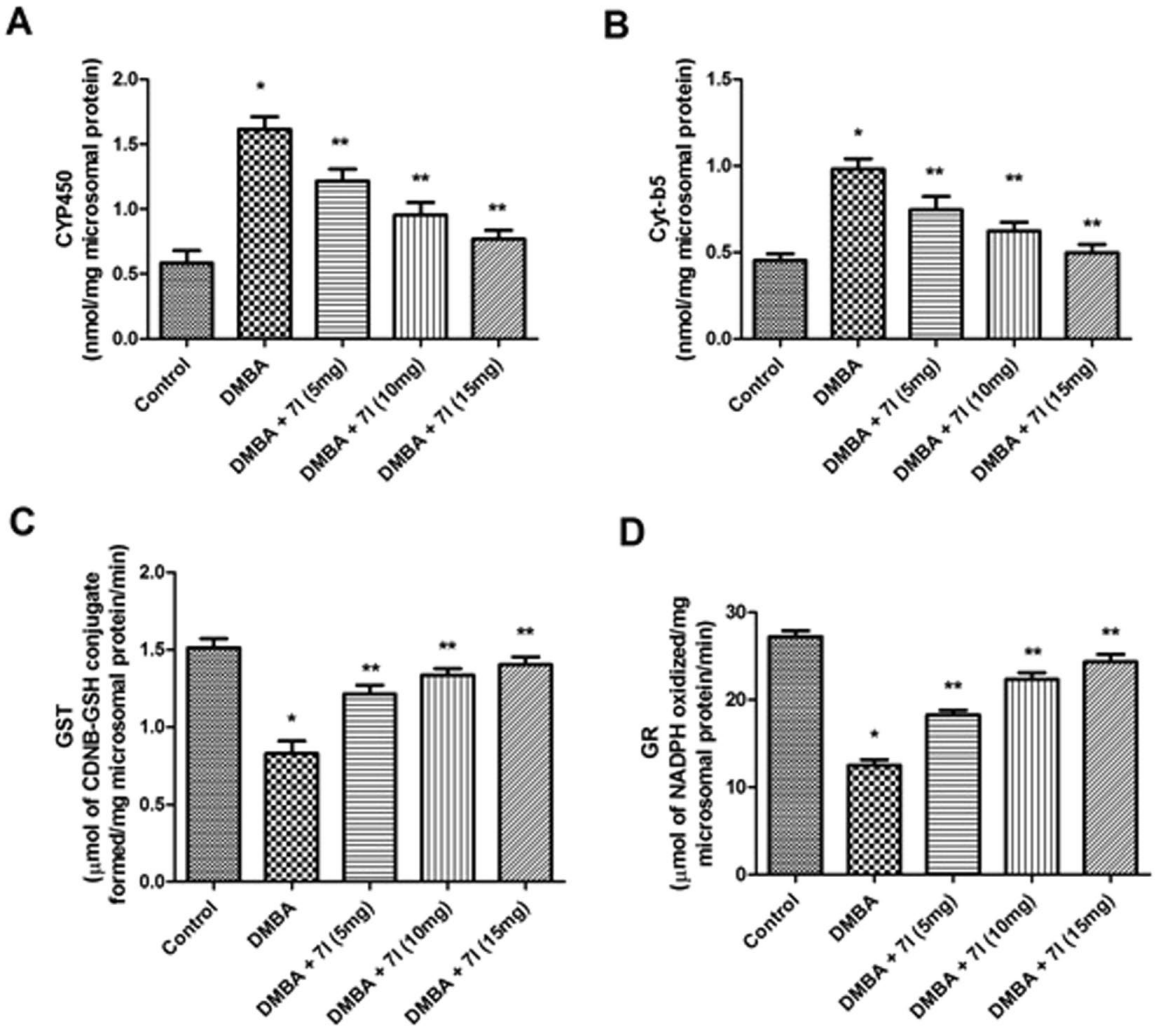
Effect of compound 7l on the level of biotransformation enzymes in liver microsomes of different treated animals. *P < 0.05 is considered as significant, **P < 0.01 is considered as very significant.

**Figure 13:**
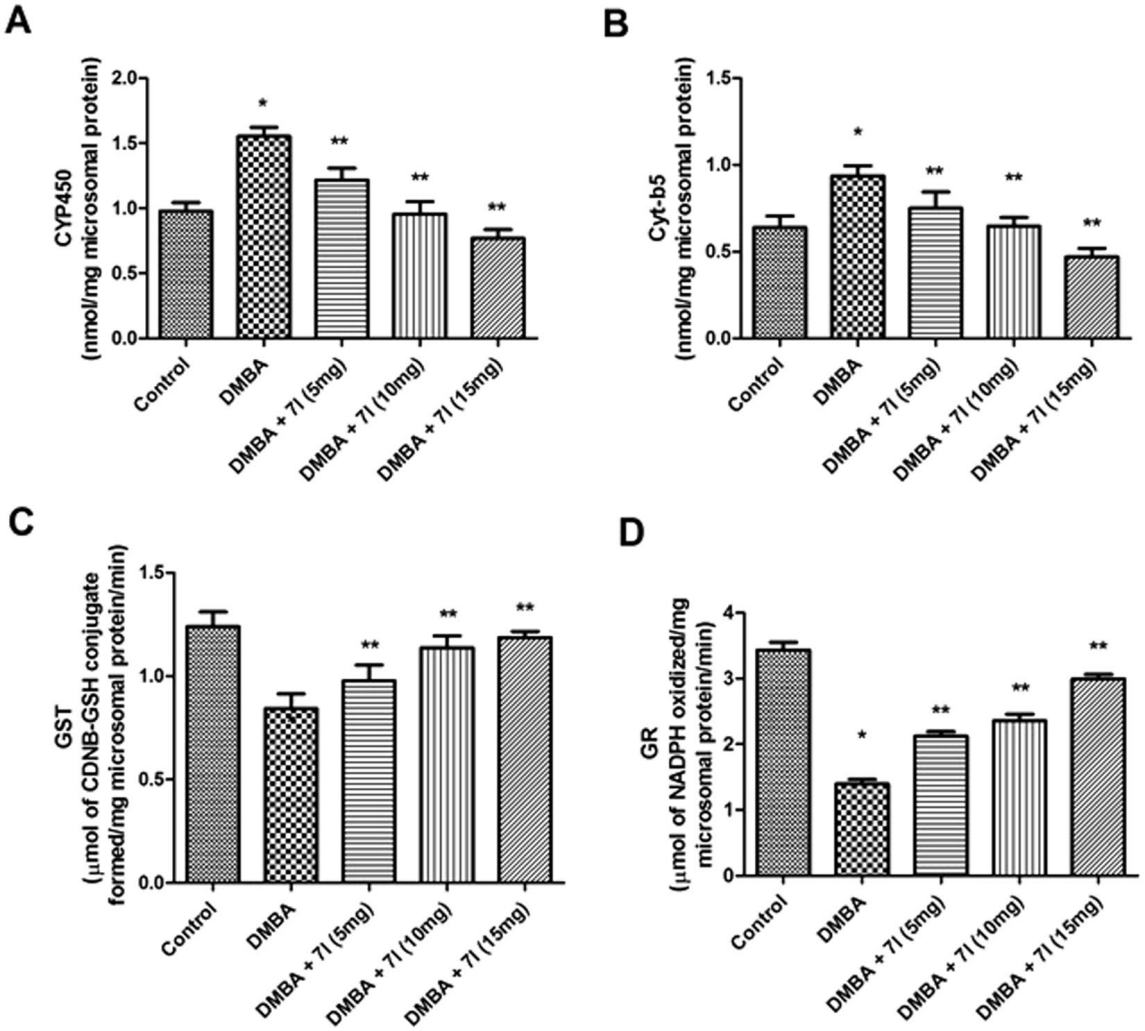
Effect of compound 7l on the level of biotransformation enzymes in mammary tissues of different treated animals. *P < 0.05 is considered as significant, **P < 0.01 is considered as very significant.

The metabolic enzymes are well known for their ability to clear the metabolic by-products generated by the cancer causing agents. In a classical metabolic pathway of the DMBA, it has been found that, CYP450 dependent oxygenases, including other Phase I enzymes causes metabolic degradation of the DMBA into reactive species which are indicated to be responsible for cancer inducing effect. Whereas, the enzymes involved in Phase II metabolic process, such as, GST and GR causes conversion of DMBA to water soluble conjugates which will be easily cleared-off and acts as a protective mechanism to prevent its cancer inducing effect. Consequently, it is imperative to determine the effect of compound **7l** on these enzymes in different treated group to determine its beneficial effect against mammary tumour. The Figs 12 and 13 clearly showed the expression of various biotransformation enzymes in the liver and mammary tissues of the treated and control animals, for instance, phase I (CYP450, Cyt-b5) and phase II (GST, GR). It has been found that, in DMBA treated group, the level of CYP450 and Cyt-b5 were found to be significantly elevated as compared to the control. Whereas, the level of Phase II enzymes, e.g. GST and GR were found to be significantly reduced. In the case of **7l** treated animals, the marked decline in the activity was reported in the concentration of Phase I enzymes (CYP450 and Cyt-b5). Moreover, a significant upsurge was reported in the concentration of both GST and GR. From the study, it has been corroborated that, compound **7l** was significantly effective in modulating the concentration of both Phase I and Phase II enzymes in dose-dependent manner. These results were also found in accordance with earlier studies, which may be indicated as possible mechanism of compound **7l** to exert potent anti-breast cancer activity.

#### Effect of compound 7**l** on the EGFR activation and downstream signalling

**Figure 14:**
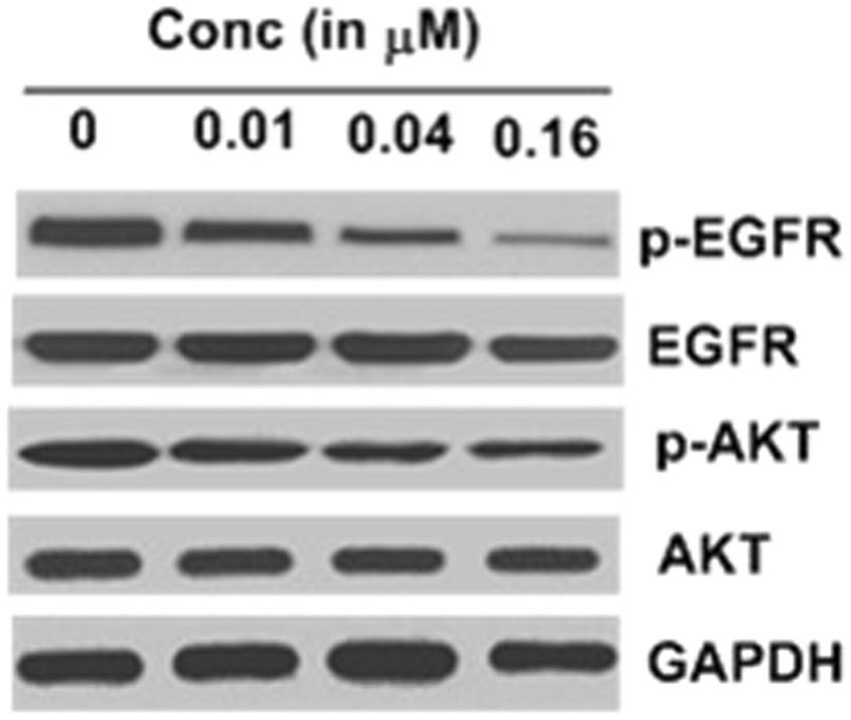
Effect of compound **7l** on the p-EGFR, EGFR, p-Akt and Akt as determined by western blot assay.

The extensive analysis of pharmacological activity of compound **7l** in both *in-vitro* and *in-vivo* studies together with docking analysis as presented above showed the excellent inhibitory activity against breast cancer *via* inhibition of EGFR-TK. However, despite of excellent inhibitory activity of **7l** against the EGFR-TK, its mechanism behind this phenomena has not been elucidate till now, thus, in this part of the study, we are interested in exploring the effect of compound **7l** on the activation and downstream signalling pathway of the EGFR. Towards this, the effect of **7l** has been determined on the expression of p-EGFR and EGFR, p-Akt and Akt levels of MCF-7 by immunoblotting experiments. Results as presented in Fig. 14 confirmed the effect of **7l** on the expression of p-EGFR and p-Akt. It has been found that, the level of both p-EGFR and p-Akt were significantly reduced in concentration-dependent manner under influence of **7l**. Results clearly indicate the ability of **7l** to inhibit the phosphorylation of EGFR which in turn decreases Akt (the downstream target protein of p-EGFR).

## Conclusion

A novel series of hybrid analogues of monastrol-1,3,5-triazine were designed and developed *via* three-component, one-pot synthesis and proved as effective anticancer agent via inhibition of EGFR-TK along with excellent *in-vivo* activity. These molecules will certainly hold a promise for the future drug development initiative.

## Experimental

### Chemistry

Melting points of the synthesized compounds was determined in an open capillary tube Hicon Melting point apparatus and are uncorrected. Thin layer chromatography (TLC) was performed on silica gel-G coated plates to detect the completion of reaction. The diverse mobile phase was selected in different proportion according to the assumed polarity of the products. The spots was visualised by exposure to the Iodine vapour. Infra-Red (IR) spectra were recorded in KBr on Biored FTs spectrophotometer and the reported wave numbers are given in cm^−1^. ^1^H NMR spectra were recorded in DMSO on Bruker Model D9RX-400MHz spectrometer. Chemical shifts were reported as δ (ppm) relative to TMS as internal standard. Mass spectra were obtained on VG-AUTOSPEC spectrometer equipped with electrospray ionization (ESI) sources.

General Procedure

Step 1: The synthesis of the various intermediates was performed in accordance with the earlier reported procedure^28^.

Step 2: Multicomponent one-pot synthesis of target derivatives, 7 (a–o)

A solution of an appropriate ethyl acetoacetate (1.2 mmol), corresponding aldehyde (1.0 mmol), urea or thiourea (1.2 mmol) in ethanol (5 ml) was heated under reflux in the presence of a catalytic amount of Bi(NO_3_)_3_ (10 mol%) till the completion of reaction as indicated by TLC. The reaction mixture was washed thoroughly with water, filtered and further purified by column chromatography to afford pure products. Elemental analysis of C, H and N was performed on a Vario EL III CHNOS elemental analyzer. The spectral data of the compounds are given below.

*Ethyl 1-(4,6-bis(phenylamino)-1,3,5-triazin-2-yl)-6-methyl-4-phenyl-2-thioxo-1,2,3,4-tetrahydropyrimidine-5-carboxylate 7a*

Yield: 68%; M.p: 225–227 °C; MW: 537.64; R_*f*_: 0.63; FTIR (ν_max_; cm^−1^ KBr): 3292 (N–H _secondary_), 3058 (C–H _broad_), 1716 (C=O), 1688–1652 (C=N _aromatic_), 1653 (C=C), 1368–1052 (C-N _aromatic_), 1272 (C=S), 1085 (CO _stretching_), 693, 548; ^1^H NMR (400 MHz, DMSO, TMS) δ ppm: 7.52–7.51 (m, 4 H, 4 × CH, Ar-H), 7.31–7.30 (m, 2 H, 2 × CH, Ar-H), 7.26–7.20 (m, 7H, 7×CH, Ar-H), 6.81–6.79 (m, 2H, 2×CH, Ar-H), 4.38 (q, 2 H *J* = 7.08 Hz, CH_2_ oxoacetate), 4.28 (s, 1H, pyrimidin), 3.87 (br,s, 2H, 2 × NH), 2.56 (s, 3H, CH_3_ pyrimidin), 1.89 (s, 1H, NH pyrimidin), 1.48 (t, 3H, *J* = 6.93 Hz, CH_3_ oxoacetate); ^13^C NMR (100 MHz, DMSO) δ ppm: 176.14, 168.73, 167.39, 167.28, 158.42, 143.32, 138.96, 129.52, 128.53, 126.92, 126.52, 122.42, 117.82, 104.28, 61.82, 58.68, 18.16, 14.23; Mass: 538.68 (M + H)^+^; Elemental analysis for C_29_H_27_N_7_O_2_S: Calculated: C, 64.79; H, 5.06; N, 18.24. Found: C, 64.81; H, 5.04; N, 18.27.

*Ethyl 1-(4,6-bis((4-nitrophenyl)amino)-1,3,5-triazin-2-yl)-6-methyl-2-thioxo-4-(p-tolyl)-1,2,3,4-tetrahydropyrimidine-5-carboxylate 7b*

Yield: 63%; M.p: 183–185 °C; MW:641.66; R_*f*_: 0.48; FTIR (ν_max_; cm^−1^ KBr): 3286 (N–H _secondary_), 3046 (C–H _broad_), 1712 (C=O), 1672–1637 (C=N _aromatic_), 1605 (C=C), 1527 (NO_2_), 1347–1023 (C-N _aromatic_), 1276 (C=S), 1093 (CO stretching), 610, 558; ^1^H NMR (400 MHz, DMSO, TMS) δ ppm: 8.12–8.08 (m, 4 H, 4 × CH, Ar-H), 7.34–7.31 (m, 4 H, 4 × CH, Ar-H), 6.89 (m, 4H, 4 × CH, Ar-H), 4.32 (s, 3H, CH_3_ pyrimidin), 4.12 (s, 1H, CH pyrimidin), 4.38 (q, 2H *J* = 7.08 Hz, CH_2_ oxoacetate), 3.85 (br,s, 2H, 2 × NH), 2.43 (s, 3H, CH_3_), 1.89 (s, 1H, NH pyrimidin), 1.42 (t, 3H, *J* = 6.94 Hz, CH_3_ oxoacetate); ^13^C NMR (100 MHz, DMSO) δ ppm: 176.23, 168.92, 167.32, 167.21, 158.43, 145.23, 140.31, 137.9, 136.40, 128.82, 126.90, 124.72, 119.21, 104.20, 61.80, 58.40, 21.36, 18.46, 14.21; Mass: 642.65 (M + H)^+^; Elemental analysis for C_30_H_27_N_9_O_6_S: Calculated: C, 56.15; H, 4.24; N, 19.65. Found: C, 56.18; H, 4.26; N, 19.68.

*Ethyl 1-(4,6-bis((2-nitrophenyl)amino)-1,3,5-triazin-2-yl)-6-methyl-2-thioxo-4-(p-tolyl)-1,2,3,4-tetrahydropyrimidine-5-carboxylate 7c*

Yield: 68%; M.p: 232–234 °C; MW: 641.66; R_*f*_: 0.54; FTIR (ν_max_; cm^−1^ KBr): 3288 (N–H _secondary_), 3048 (C–H _broad_), 1715 (C=O), 1674–1639 (C=N _aromatic_), 1611 (C=C), 1528 (NO_2_), 1347–1026 (C-N _aromatic_), 1278 (C=S), 1091 (CO _stretching_), 628, 562; ^1^H NMR (400 MHz, DMSO, TMS) δ ppm: 8.08 (m, 2H, 2 × CH, No2- Ar-H), 7.59–7.51 (m, 4H, 4 × CH, No2-Ar-H), 7.34 (s, 1H Ar-H), 7.13–7.11 (m, 3H, 3 × CH, Ar-H), 6.89 (m, 2H, 2 × CH, Ar-H), 4.52 (s, 1H, CH pyrimidin), 4.46 (q, 2H *J* = 7.06 Hz, CH_2_ oxoacetate), 3.87 (br,s, 1H, NH), 2.36 (s, 3H, CH_3_ Ar), 2.13 (s, 3H, CH_3_ pyrimidin), 1.88 (s, 1H, NH), 1.36 (t, 3H *J* = 6.89 Hz, CH_3_ oxoacetate); ^13^C NMR (100 MHz, DMSO) δ ppm: 176.21, 168.82, 167.36, 167.25, 158.42, 144.60, 140.32, 137.14, 136.43, 129.10, 128.82, 126.76, 125.84, 119.64, 110.58, 104.24, 61.71, 58.60, 21.28, 18.52, 14.21; Mass: 642.68 (M + H)^+^; Elemental analysis for C_30_H_27_N_9_O_6_S: Calculated: C, 56.15; H, 4.24; N, 19.65. Found: C, 56.18; H, 4.25; N, 19.64.

*Ethyl 1-(4,6-bis((3-chlorophenyl)amino)-1,3,5-triazin-2-yl)-4-(4-hydroxyphenyl)-6-methyl-2-thioxo-1,2,3,4-tetrahydropyrimidine-5-carboxylate 7d*

Yield: 71%; M.p: 161–163 °C; MW: 622.52; R_*f*_: 0.42; FTIR (ν_max_; cm^−1^ KBr): 3478 (OH), 3282 (N–H _secondary_), 3053 (C–H _broad_), 1721 (C=O), 1674–1642 (C=N _aromatic_), 1618 (C=C), 1349–1023 (C-N _aromatic_), 1268 (C=S), 1082 (CO _stretching_) 1014 (C-Cl _stretching_), 678, 549; ^1^H NMR (400 MHz, DMSO, TMS) δ ppm: 7.68–7.61 (m, 2H, 2 × CH, Ar-H), 7.14–7.08 (m, 4H, 4 × CH, Ar-H), 6.87–6.82 (m, 4H, 4 × CH, Ar-H), 6.63–6.58 (m, 2H, 2 × CH, Ar-H), 5.46 (s, 1H, Ar-OH), 4.48 (q, 2H *J* = 7.06 Hz, CH2 oxoacetate), 4.08 (s, 1H, pyrimidin), 3.89 (br,s, 2H, 2 × NH), 2.34 (s, 3H, CH_3_ pyrimidin), 1.94 (s, 1H, NH), 1.36 (t, 3H *J* = 6.89 Hz, CH_3_ oxoacetate); ^13^C NMR (100 MHz, DMSO) δ ppm: 176.24, 168.80, 167.32, 167.24, 158.42, 156.54, 143.84, 135.92, 135.10, 130.90, 126.74, 122.46, 116.68, 115.90, 115.74, 104.26, 61.78, 58.48, 18.24, 14.26; Mass: 623.56 (M + H)^+^; Elemental analysis for C_29_H_25_Cl_2_N_7_O_3_S: Calculated: C, 55.95; H, 4.05; N, 15.75. Found: C, 55.98; H, 4.09; N, 15.77.

*Ethyl 1-(4,6-bis((3-bromophenyl)amino)-1,3,5-triazin-2-yl)-4-(2-hydroxyphenyl)-6-methyl-2-thioxo-1,2,3,4-tetrahydropyrimidine-5-carboxylate 7e*

Yield: 69%; M.p: 245–247 °C; MW: 711.43; R_*f*_: 0.48; FTIR (ν_max_; cm^−1^ KBr): 3472 (OH), 3286 (N–H _secondary_), 3048 (C–H _broad_), 1716 (C=O), 1676–1638 (C=N _aromatic_), 1621 (C=C), 1342–1018 (C-N _aromatic_), 1271 (C=S), 1086 (CO _stretching_) 916 (C-Br _stretching_), 669, 538; ^1^H NMR (400 MHz, DMSO, TMS) δ ppm: 7.57–7.54 (m, 2 H, 2 × CH, Ar-H), 7.09–7.06 (m, 2H, 2 × CH, Ar-H), 6.96–6.92 (m, 4H, 4 × CH, Ar-H), 6.76–6.64 (m, 4H, 4 × CH, Ar-H), 5.44 (s, 1H, Ar-OH), 4.46 (q, 2H *J* = 7.06 Hz, CH_2_ oxoacetate), 4.12 (s, 1H, pyrimidin), 3.94 (br,s, 2H, 2 × NH), 2.32 (s, 3H, CH_3_ pyrimidin), 1.96 (s, 1H, NH pyrimidin), 1.38 (t, 3 H *J* = 6.91 Hz, CH_3_ oxoacetate); ^13^C NMR (100 MHz, DMSO) δ ppm: 176.10, 168.84, 167.30, 167.21, 158.40, 154.03, 144.68, 130.69, 128.42, 128.10, 123.86, 122.74, 121.54, 121.10, 116.86, 115.84, 115.52, 104.23, 61.70, 52.41, 18.23, 14.16; Mass: 712.46 (M + H)^+^; Elemental analysis for C_29_H_25_Br_2_N_7_O_3_S: Calculated: C, 48.96; H, 3.54; N, 13.78. Found: C, 48.98; H, 3.52; N, 13.81.

*Ethyl 1-(4,6-bis((2-chlorophenyl)amino)-1,3,5-triazin-2-yl)-4-(4-methoxyphenyl)-6-methyl-2-thioxo-1,2,3,4-tetrahydropyrimidine-5-carboxylate 7f*

Yield: 72%; M.p: 91–93 °C; MW: 636.55; R_*f*_: 0.48; FTIR (ν_max_; cm^−1^ KBr): 3286 (N–H _secondary_), 3052 (C–H _broad_), 1721 (C = O), 1672–1646 (C = N _aromatic_), 1614 (C = C), 1348–1026 (C-N _aromatic_),1264 (C = S), 1084 (COO _stretching_) 1024 (C-Cl _stretching_), 676, 538; ^1^H NMR (400 MHz, DMSO, TMS) δ ppm: 8.08–8.04 (m, 2 H, 2 × CH, Ar-H), 7.45–7.38 (m, 4 H, 4 × CH, Ar-H), 7.12–7.08 (m, 2 H, 2 × CH, Ar-H), 6.85–6.78 (m, 4 H, 4 × CH, Ar-H), 4.38 (q, 2 H *J* = 7.04 Hz, CH_2_ oxoacetate), 4.18 (s, 1 H, pyrimidin), 3.89 (br,s, 2 H, 2 × NH), 3.74 (s, 3 H, Ar-OCH_3_), 2.42 (s, 3 H, CH_3_ pyrimidin), 1.92 (s, 1 H, NH pyrimidin), 1.32 (t, 3 H* J* = 6.93 Hz, CH_3_ oxoacetate); ^13^C NMR (100 MHz, DMSO) δ ppm: 176.12, 168.94, 167.34, 167.23, 158.62, 158.41, 136.42, 135.61, 130.72, 127.60, 125.72, 125.41, 122.83, 122.24, 114.12, 104.24, 61.71, 58.65, 55.82, 18.30, 14.24; Mass: 637.58 (M + H)^+^; Elemental analysis for C_30_H_27_Cl_2_N_7_O_3_S: Calculated: C, 56.61; H, 4.28; N, 15.40. Found: C, 56.66; H, 4.32; N, 15.42.

*Ethyl 1-(4,6-bis((3-fluorophenyl)amino)-1,3,5-triazin-2-yl)-4-(2-methoxyphenyl)-6-methyl-2-thioxo-1,2,3,4-tetrahydropyrimidine-5-carboxylate 7g*

Yield: 63%; M.p: 132–135 °C; MW: 603.64; R_*f*_: 0.62; FTIR (ν_max_; cm^−1^ KBr): 3283 (N–H _secondary_), 3056 (C–H _broad_), 1723 (C = O), 1678–1651 (C = N _aromatic_), 1618 (C = C), 1349–1028 (C-N _aromatic_),1262 (C = S), 1084 (CO _stretching_), 978 (C-F _stretching_), 685, 548; ^1^H NMR (400 MHz, DMSO, TMS) δ ppm: 7.76–7.74 (m, 2 H, 2 × CH, Ar-H), 7.38–7.26 (m, 4 H, 4 × CH, Ar-H), 7.02–6.89 (m, 3 H, 3 × CH, Ar-H), 6.82 (d, 1 H *J* = 5.45 Hz, Ar-H), 6.58–6.56 (m, 2 H, 2 × CH, Ar-H), 4.32 (q, 2 H *J* = 7.06 Hz, CH_2_ oxoacetate), 4.24 (s, 1 H, pyrimidin), 3.87 (br,s, 2 H, 2 × NH), 3.81 (s, 3 H, Ar-OCH_3_), 2.46 (s, 3 H, CH_3_ pyrimidin), 1.98 (s, 1 H, NH pyrimidin), 1.29 (t, 3 H* J* = 6.96 Hz, CH_3_ oxoacetate); ^13^C NMR (100 MHz, DMSO) δ ppm: 176.12, 168.86, 167.38, 167.21, 163.72, 158.44, 156.58, 144.18, 131.12, 127.89, 127.73, 121.24, 120.87, 113.46, 112.38, 110.54, 104.86, 104.21, 61.76, 56.14, 52.64, 18.20, 14.26; Mass: 604.65 (M + H)^+^; Elemental analysis for C_30_H_27_F_2_N_7_O_3_S: Calculated: C, 59.69; H, 4.51; N, 16.24. Found: C, 59.68; H, 4.52; N, 16.26.

*Ethyl 1-(4,6-bis((4-nitrophenyl)amino)-1,3,5-triazin-2-yl)-4-(4-chlorophenyl)-6-methyl-2-thioxo-1,2,3,4-tetrahydropyrimidine-5-carboxylate 7h*

Yield: 64%; M.p: 146–147 °C; MW: 662.08; R_*f*_: 0.65; FTIR (ν_max_; cm^−1^ KBr): 3289 (N–H _secondary_), 3052 (C–H _broad_), 1721 (C = O), 1678–1656 (C = N _aromatic_), 1621 (C = C), 1528 (NO_2_), 1356–1036 (C-N _aromatic_), 1268 (C = S), 1089 (CO _stretching_) 1019 (C-Cl _stretching_), 689, 552; ^1^H NMR (400 MHz, DMSO, TMS) δ ppm: 8.01–7.98 (m, 4 H, 4 × CH, Ar-H), 7.38–7.36 (m, 4 H, 4 × CH, Ar-H), 6.89–6.87 (m, 4 H, 4 × CH, Ar-H), 4.36 (q, 2 H *J* = 7.06 Hz, CH_2_ oxoacetate), 4.23 (s, 1 H, pyrimidin), 3.89 (br,s, 2 H, 2 × NH), 2.49 (s, 3 H, CH_3_ pyrimidin), 1.94 (s, 1 H, NH pyrimidin), 1.36 (t, 3 H* J* = 6.84 Hz, CH_3_ oxoacetate); ^13^C NMR (100 MHz, DMSO) δ ppm: 176.18, 168.82, 167.39, 167.22, 158.39, 145.21, 141.46, 137.92, 132.38, 128.64, 126.17, 124.74, 119.26, 104.24, 61.72, 58.68, 18.18, 14.26; Mass: 663.10 (M + H)^+^; Elemental analysis for C_29_H_24_ClN_9_O_6_S: Calculated: C, 52.61; H, 3.65; N, 19.04. Found: C, 52.64; H, 3.68; N, 19.02.

*Ethyl 1-(4,6-bis((2-nitrophenyl)amino)-1,3,5-triazin-2-yl)-4-(3-bromophenyl)-6-methyl-2-thioxo-1,2,3,4-tetrahydropyrimidine-5-carboxylate 7i*

Yield: 78%; M.p.: 185–186 °C; MW: 706.53; R_*f*_: 0.59; FTIR (ν_max_; cm^−1^ KBr): 3292 (N–H _secondary_), 3058 (C–H _broad_), 1716 (C = O), 1682–1658 (C = N _aromatic_), 1628 (C = C), 1518 (NO_2_), 1355–1032 (C-N _aromatic_),1258 (C = S), 1085 (CO _stretching_) 985 (C-Br _stretching_), 695, 558; ^1^H NMR (400 MHz, DMSO, TMS) δ ppm: 8.08–8.06 (m, 2 H, 2 × CH, Ar-H), 7.64–7.62 (m, 2 H, 2 × CH, Ar-H), 7.42–7.38 (m, 4 H, 4 × CH, Ar-H), 7.22–7.19 (m, 2 H, 2 × CH, Ar-H), 6.89–6.87 (m, 2 H, Ar-H), 4.38 (q, 2 H *J* = 7.04 Hz, CH_2_ oxoacetate), 4.22 (s, 1 H, pyrimidin), 3.84 (br,s, 2 H, 2 × NH), 2.51 (s, 3 H, CH_3_ pyrimidin), 1.96 (s, 1 H, NH pyrimidin), 1.42 (t, 3 H* J* = 6.94 Hz, CH_3_ oxoacetate); ^13^C NMR (100 MHz, DMSO) δ ppm: 176.21, 168.86, 167.36, 167.23, 158.47, 145.56, 144.68, 137.12, 131.78, 129.94, 129.21, 129.00, 125.94, 125.38, 122.92, 119.67, 110.58, 104.21, 61.72, 57.93, 18.21, 14.16; Mass: 707.56 (M + H)^+^; Elemental analysis for C_29_H_24_BrN_9_O_6_S: Calculated: C, 49.30; H, 3.42; N, 17.84. Found: C, 49.32; H, 3.41; N, 17.86.

*Ethyl 1-(4,6-bis((4-chlorophenyl)amino)-1,3,5-triazin-2-yl)-4-(2-chlorophenyl)-6-methyl-2-thioxo-1,2,3,4-tetrahydropyrimidine-5-carboxylate 7j*

Yield: 59%; M.p: 112–114 °C; MW: 640.97; R_*f*_: 0.49; FTIR (ν_max_; cm^−1^ KBr): 3296 (N–H _secondary_), 3052 (C–H _broad_), 1726 (C=O), 1684–1652 (C=N _aromatic_), 1634 (C=C), 1358–1026 (C-N _aromatic_),1252 (C=S), 1078 (CO _stretching_), 1019 (C-Cl _stretching_), 698, 565; ^1^H NMR (400 MHz, DMSO, TMS) δ ppm: 7.73–7.68 (m, 5H, 5 × CH, Ar-H), 7.24–7.18 (m, 7H, 7 × CH, Ar-H), 4.36 (q, 2H *J* = 7.02 Hz, CH_2_ oxoacetate), 4.24 (s, 1H, pyrimidin), 3.86 (br,s, 2H, 2 × NH), 2.56 (s, 3H, CH_3_ pyrimidin), 1.94 (s, 1H, NH pyrimidin), 1.47 (t, 3H *J* = 6.92 Hz, CH_3_ oxoacetate); ^13^C NMR (100 MHz, DMSO) δ ppm: 176.11, 168.89, 167.32, 167.24, 158.42, 142.86, 137.18, 132.28, 129.64, 128.60, 128.34, 128.12, 127.78, 126.62, 122.17, 104.24, 61.72, 53.58, 18.21, 14.18; Mass: 641.98 (M + H)^+^; Elemental analysis for C_29_H_24_Cl_3_N_7_O_2_S: Calculated: C, 54.34; H, 3.77; N, 15.30. Found: C, 54.38; H, 3.78; N, 15.32.

*Ethyl 1-(4,6-bis((3-bromophenyl)amino)-1,3,5-triazin-2-yl)-6-methyl-4-(4-nitrophenyl)-2-thioxo-1,2,3,4-tetrahydropyrimidine-5-carboxylate 7k*

Yield: 64%; M.p: 167–165 °C; MW: 740.43; R_*f*_: 0.58; FTIR (ν_max_; cm^−1^ KBr): 3298 (N–H _secondary_), 3048 (C–H _broad_), 1723 (C=O), 1684–1656 (C=N _aromatic_), 1638 (C=C), 1526 (NO_2_), 1361–1028 (C-N _aromatic_),1257 (C=S), 1075 (CO _stretching_), 987 (C-Br _stretching_), 678, 569; ^1^H NMR (400 MHz, DMSO, TMS) δ ppm: 8.06–8.05 (m, 2 H, 2 × CH, Ar-H), 7.54–7.49 (m, 4 H, 4 × CH, Ar-H), 6.96–6.94 (m, 4 H, 4 × CH, Ar-H), 6.64–6.62 (m, 2H, 2 × CH, Ar-H), 4.34 (q, 2H *J* = 7.08 Hz, CH_2_ oxoacetate), 4.34 (s, 1H, pyrimidin), 3.87 (br,s, 2H, 2 × NH), 2.58 (t, 3H *J* = 6.88 Hz, CH_3_ pyrimidin), 1.92 (s, 1H, NH pyrimidin), 1.38 (s, 3H, CH_3_ oxoacetate); ^13^C NMR (100 MHz, DMSO) δ ppm: 176.16, 168.86, 167.32, 167.12, 158.45, 149.46, 145.92, 144.67, 130.62, 128.32, 123.92, 121.62, 116.81, 115.54, 104.28, 61.79, 58.62, 18.16, 14.22; Mass: 741.48 (M + H)^+^; Elemental analysis for C_29_H_24_Br_2_N_8_O_4_S: Calculated: C, 47.04; H, 3.27; N, 15.13. Found: C, 47.01; H, 3.29; N, 15.11.

*Ethyl 1-(4,6-bis((3-fluorophenyl)amino)-1,3,5-triazin-2-yl)-4-(4-fluorophenyl)-6-methyl-2-thioxo-1,2,3,4-tetrahydropyrimidine-5-carboxylate 7l*

Yield: 68%; M.p: 171–172 °C; MW: 591.61; R_*f*_: 0.54; FTIR (ν_max_; cm^−1^ KBr): 3294 (N–H _secondary_), 3048 (C–H _broad_), 1721 (C=O), 1686–1654 (C=N _aromatic_), 1642 (C=C), 1367–1038 (C-N _aromatic_),1259 (C=S), 1078 (CO _stretching_), 978 (C-F _stretching_), 689, 572; ^1^H NMR (400 MHz, DMSO, TMS) δ ppm: 7.72–7.70 (m, 2H, 2 × CH, Ar-H), 7.40–7.38 (m, 2H, 2 × CH, Ar-H), 7.21–7.14 (m, 6H, 6 × CH, Ar-H), 6.63–6.62 (m, 2H, 2 × CH, Ar-H), 4.38 (q, 2H *J* = 7.04 Hz, CH_2_ oxoacetate), 4.32 (s, 1H, pyrimidin), 3.87 (br,s, 2H, 2 × NH), 2.58 (s, 3H, CH_3_ pyrimidin), 1.87 (s, 1H, NH pyrimidin), 1.42 (t, 3H *J* = 7.04 Hz, CH_3_ oxoacetate); ^13^C NMR (100 MHz, DMSO) δ ppm: 176.24, 168.81, 167.38, 167.23, 163.79, 160.92, 158.42, 144.18, 138.92, 131.12, 128.50, 115.32, 113.42, 110.52, 104.86, 104.22, 61.72, 58.68, 18.18, 14.24; Mass: 592.64 (M + H)^+^; Elemental analysis for C_29_H_24_F_3_N_7_O_2_S: Calculated: C, 58.88; H, 4.09; N, 16.57. Found: C, 58.90; H, 4.11; N, 16.56.

*Ethyl 1-(4,6-bis((3-chlorophenyl)amino)-1,3,5-triazin-2-yl)-4-(4-fluorophenyl)-6-methyl-2-thioxo-1,2,3,4-tetrahydropyrimidine-5-carboxylate 7m*

Yield: 72%; M.p: 153–155 °C; MW: 624.52; R_*f*_: 0.63; FTIR (ν_max_; cm^−1^ KBr): 3296 (N–H _secondary_), 3042 (C–H _broad_), 1718 (C=O), 1684–1652 (C=N _aromatic_), 1648 (C=C), 1367–1042 (C-N _aromatic_),1265 (C=S), 1082 (CO _stretching_), 992 (C-Cl _stretching_), 972 (C-F _stretching_), 678, 578; ^1^H NMR (400 MHz, DMSO, TMS) δ ppm: 7.78–7.76 (m, 2H, 2 × CH, Ar-H), 7.45–7.44 (m, 2H, 2 × CH, Ar-H), 7.28–7.21 (m, 6H, 6 × CH, Ar-H), 7.07–7.06 (m, 2H, 2 × CH, Ar-H), 4.35 (q, 2H *J* = 7.05 Hz, CH_2_ oxoacetate), 4.28 (s, 1H, pyrimidin), 3.84 (br,s, 2H, 2 × NH), 2.58 (s, 3 H, CH_3_ pyrimidin), 1.89 (s, 1H, NH pyrimidin), 1.48 (t, 3H *J* = 6.94 Hz, CH_3_ oxoacetate); ^13^C NMR (100 MHz, DMSO) δ ppm: 176.14, 168.86, 167.32, 167.22, 160.92, 158.84, 143.82, 138.92, 135.18, 130.92, 128.52, 122.34, 116.72, 115.92, 115.36, 104.24, 61.74, 58.68, 18.21, 14.25; Mass: 625.53 (M + H)^+^; Elemental analysis for C_29_H_24_Cl_2_FN_7_O_2_S: Calculated: C, 55.77; H, 3.87; N, 15.70. Found: C, 55.79; H, 3.91; N, 15.72.

*Ethyl 1-(4,6-bis(p-tolylamino)-1,3,5-triazin-2-yl)-6-methyl-2-thioxo-4-(o-tolyl)-1,2,3,4-tetrahydropyrimidine-5-carboxylate 7n*

Yield: 61%; M.p: 205–207 °C; MW: 579.72; R_*f*_: 0.52; FTIR (ν_max_; cm^−1^ KBr): 3292 (N–H _secondary_), 3048 (C–H _broad_), 1719 (C=O), 1686–1658 (C=N _aromatic_), 1652 (C=C), 1418 (-CH3), 1365–1048 (C-N _aromatic_), 1263 (C=S), 1087 (CO _stretching_), 696, 512; ^1^H NMR (400 MHz, DMSO, TMS) δ ppm: 7.33–7.32 (m, 4H, 4 × CH, Ar-H), 7.18 (s, 1H, 1 × CH, Ar-H), 7.08–7.05 (m, 7H, 7 × CH, Ar-H), 4.38 (q, 2H *J* = 7.08 Hz, CH_2_ oxoacetate), 4.32 (s, 1H, pyrimidin), 3.87 (br,s, 2H, 2 × NH), 2.57 (s, 3H, CH_3_ pyrimidin), 2.23–2.21 (s, 9H, 3 × CH_3_ Ar-CH_3_), 1.89 (s, 1 H, NH pyrimidin), 1.48 (t, 3H *J* = 6.93 Hz, CH_3_ oxoacetate); ^13^C NMR (100 MHz, DMSO) δ ppm: 176.34, 168.81, 167.38, 167.21, 158.87, 142.72, 135.98, 134.78, 131.12, 130.28, 129.82, 126.62, 125.58, 124.92, 120.37, 104.26, 61.72, 56.13, 21.31, 19.28, 18.16, 14.24; Mass: 580.74 (M + H)^+^; Elemental analysis for C_32_H_33_N_7_O_2_S: Calculated: C, 66.30; H, 5.74; N, 16.91. Found: C, 66.32; H, 5.78; N, 16.92.

*Ethyl 1-(4,6-bis(p-tolylamino)-1,3,5-triazin-2-yl)-4-(4-hydroxyphenyl)-6-methyl-2-thioxo-1,2,3,4-tetrahydropyrimidine-5-carboxylate 7o*

Yield: 48%; M.p: 131–133 °C; MW: 581.69; R_*f*_: 0.58; FTIR (ν_max_; cm^−1^ KBr): 3296 (N–H _secondary_), 3051 (C–H _broad_), 1723 (C=O), 1683–1654 (C=N _aromatic_), 1658 (C=C), 1365–1057 (C-N _aromatic_),1268 (C=S), 1093 (CO _stretching_), 699, 538; ^1^H NMR (400 MHz, DMSO, TMS) δ ppm: 7.33–7.31 (m, 4H, 4 × CH, Ar-H), 7.06–7.04 (m, 6H, 6 × CH, Ar-H), 6.67–6.65 (m, 2H, 2 × CH, Ar-H), 5.28 (s, 1H, Ar-OH), 4.37 (q, 2H *J* = 7.03 Hz, CH_2_ oxoacetate), 4.36 (s, 1H, pyrimidin), 3.89 (br,s, 2H, 2 × NH), 2.59 (s, 3H, CH_3_ pyrimidin), 2.23–2.21 (s, 6H, 2 × CH_3_ Ar-CH_3_), 1.89 (s, 1H, NH pyrimidin), 1.48 (t, 3H *J* = 6.91 Hz, CH_3_ oxoacetate); ^13^C NMR (100 MHz, DMSO) δ ppm: 176.24, 168.87, 167.32, 167.21, 158.46, 156.42, 136.52, 135.92, 131.23, 129.82, 126.12, 120.32, 115.78, 104.22, 61.76, 58.63, 21.38, 18.12, 14.23; Mass: 582.69 (M + H)^+^; Elemental analysis for C_31_H_31_N_7_O_3_S: Calculated: C, 64.01; H, 5.37; N, 16.86. Found: C, 64.04; H, 5.36; N, 16.89.

### Biological Evaluation

#### In vitro Anticancer Evaluation

The target compounds were subjected to *in vitro* cytotoxicity bioassay. *In vitro* cytotoxicity was determined using a standard MTT assay with protocol appropriate for the individual test system^29–31^. The four human cancer cell lines HeLa, MCF-7, HL-60, HepG2 and MCF-12A were cultured in the MEM medium supplemented with 10% FBS, 1% glutamine and 50 mM/ml gentamicin sulfate in a CO_2_ incubator in a humidified atmosphere of 5% CO_2_ and 95% air. The test compounds were prepared prior to the experiment by dissolving in 0.1% DMSO and diluted with medium. The cells were then exposed to different concentrations of drugs (1–100 mM) in the volume of 100 mM/well. Cells in the control wells received the same volume of medium containing 0.1% DMSO. After 24 h, the medium was removed and cell cultures were incubated with 100 ml MTT reagent (1 mg/ml) for 5 h at 37 °C. The known number of cells (1.0 ± 10^5^) was incubated in a 5% CO_2_ incubator at 37 °C in the presence of different concentrations of test compounds. After 48 h of drug incubation, the MTT solution was added in each well and absorbance was recorded at 540 nm by an ELISA reader. The experiment was performed in triplicate. Cell survival was calculated as the percentage of MTT inhibition as % growth inhibition = 100 − (mean OD of individual test Group/Mean OD of each Control Group) × 100.

#### EGFR tyrosine kinase inhibitory activity

Kinase activity was determined using Kinase-Glo Plus luminescence kinase assay kit, by quantitating the amount of ATP remaining in the solution of kinase reaction^32^. The luminescent signal is correlated with the residual amount present and it was inversely related with kinase activity. The tested compounds were diluted to 100 mM in 10% DMSO, then 5 mL of the dilution was added to a 50 mL reaction. All of the enzymatic reactions were performed at 30 °C for 40 min, 50 mL of reaction mixture contains 10 mM MgCl_2_, 40 mM Tris, pH 7.4, 0.1 mg/mL BSA, 0.2 mg/mL Poly (Glu, Tyr) substrate, 10 mM ATP and EGFR. Incubate the plate for 5 min at r.t. then add 50 mL of Kinase-GloPlus Luminescence kinase assay to each reaction. ADP-Glo assay kit is the protein kinase assays used to determine IC_50_ values in which ADP generation was measured as it leads to an increase in luminescence signal. The reaction mixture was incubated in a 96-well plate at 30 °C for 30 min, after the incubation period add 25 mL of ADP-Glo reagent to terminate the assay. Shake the 96-well plate for 30 min at ambient temperature then incubate it, then add 50 mL of kinase detection reagent. Read the 96-well plate using the ADP-Glo Luminescence reader. All the assay components were added to the blank control except the substrate. By removing the blank control value you can obtain the corrected activity for each protein kinase target.

#### In Vivo Anticancer evaluation

*Chemicals*. The chemicals used in the study were purchased from Sigma-Aldrich Chemicals (USA) and were of analytical grade.

*Animal model*. The female Sprague-Dawley rats (100–120 g) were obtained from the Institutional animal house after approval from the Institutional Animal Ethics Committee. The animals were kept in suitable cages with free access to food and water under a controlled temperature (24 ± 2 °C), humidity (60 ± 10%) with alternate light/dark cycle of 12 h. The entire experimental procedures involving animals were approved by the Institutional Animal Ethics Committees (IAEC) of SHUATS, in accordance and duly approved with CPCSEA (Committee for the Purpose of Control and Supervision of Experiments on Animals, Government of India. The authors confirm that all experiments were performed in accordance with relevant guidelines and regulations of SHUATS.

*Initiation of breast carcinogenesis*. The mammary carcinogenesis was induced in experimental rat using one-time subcutaneous administration of DMBA (25 mg) in emulsified form with the help of sunflower oil and saline to each rat.

*Selection of test compound dose*. The dose of compound 7**l** was estimated using an acute toxicity study in healthy Sprague Dawley rats (6–8 weeks old). The animals were observed for 48 hours after the administration of drug for the onset of clinical or toxicological symptoms. Mortality, if any, was observed over a period of 2 weeks. It has been found that, upto 40 mg/kg, no mortality has been reported. Thus, dose of 5 mg/kg, 10 mg/kg and 15 mg/kg was selected in the present study.

*Design of experiment*. *Experimental design*. Total numbers of 30 animals were randomly divided into five groups and each group contains 6 animals. The various groups received different treatment as follows:

Group I: Untreated control

Groups II: DMBA

Group III: DMBA + 7l (5 mg/kg)

Group IV: DMBA + 7l (10 mg/kg)

Group V: DMBA + 7l (15 mg/kg)

The DMBA (s.c.; 25 mg/rat) was administered to each rats near mammary gland at the end of the first week. The test drug was administered once in a day and was continued till the end of the experimental period. At the end of 16^th^ week, the rats were fasted overnight and sacrificed by the cervical decapitation under influence of anesthesia. The blood samples were collected in and after centrifugation, the plasma was used for the further biochemical analysis. The liver and mammary tissues were excised immediately from the experimental rats and homogenized with suitable buffer, centrifuged and the resulting supernatant was used for biochemical estimations.

*Biochemical Estimation*. The remaining excised liver and mammary tissues were rinsed in ice-cold saline and known amount of the tissue were homogenized in 0.1 M Tris-HCl buffer (pH 7.4) at 4°C. The supernatant was collected as tissue homogenate and was used for the estimation of various biochemical parameters. The microsomal protein concentration was estimated by the method of Lowry *et al*.^33^; while, the concentration of plasma and mammary TBARS was estimated by the earlier reported method^34, 35^ and the concentration of plasma and mammary tissue LOOH were estimated by the previous method^36^. Moreover, the activity of the antioxidant enzyme system, such as SOD, CAT, GPX, GSH, CYP450, Cyt-b5, GST and GR activity in plasma and mammary tissue were determined by the earlier method given elsewhere^37–43^.

*Lipid profile*. The estimation of lipid parameters was performed using the lipid extracted from plasma and mammary tissues by previous reported method of ref. ^44^. Moreover, the level of TC, TG, FFA, PL, LDL, VLDL-C, HDL-C and cholesterol in the plasma and mammary tissue were approximated using the earlier reported method^45–50^.

#### Western blot analysis

After incubation with the test compound, the cells were harvested and washed with PBS, and then lysed in lysis buffer. After centrifugation at 12,000 × g for 5 min, the supernatant was collected as total protein. The concentration of the protein was determined by a protein assay kit. The protein samples were separated by 10% SDS–PAGE and subsequently electro-transferred onto a PVDF (Millipore, Bedford, MA, USA). The membrane was blocked with 5% skimmed milk for 2 h at room temperature. The blocked membrane was probed with the indicated primary antibodies overnight at 4°C, and then incubated with a horse radish peroxidase (HRP)-coupled secondary antibody. The antibodies were purchased from Cell Signaling Technology (MA, USA) Thus, the expression of p-EGFR, EGFR, p-Akt (pS473) and Akt were visualized by enhanced chemiluminescence (ECL) (Thermofischer Scientific Inc. USA) kit and band intensity was analyzed by image j software.

### Molecular docking analysis

#### EGFR-TK inhibitor

It was worthwhile to perform molecular docking of compounds to define precise key interaction with EGFR. Molecular docking analysis were undertaken to evaluate the most preferred geometry of protein–ligand complexes. In this study, we used Autodock 4.2 for molecular docking studies^51, 52^. EGFR tyrosine kinase domain with 4-anilinoquinazoline inhibitor erlotinib (ERL) (PDB ID:1M17)^53^ was considered as the target protein. The protein was prepared as a receptor for docking process by protonating^54, 55^ and the prepared protein was then loaded to MGL – AutoDock Tools to identify the binding sites of EGFR-TK domain using the co-crystal ligand and assign polar hydrogen atoms including Kollman charges to the minimized protein^56^. The program AutoGrid was used to generate the grid maps for the binding site of the protein. Grid was generated based on the amino acid residues in the active center and co-crystal ligand bound interaction points, further in order to match different interaction types like Br, Cl, F, SA, OA for all ligands, the grid generation parameter was manually updated to generate the custom grid maps for the protein binding site. Binding site includes Met 769, Gly 772, Leu 694, Leu 768, Lys 721, Thr 766, Thr 830 and Asp 831 amino acid residues in protein kinases ATP-binding region and Tyrosine protein kinases specific active-site regions.The ligands 7a-7o, and erlotinib (ERL) was drawn using Marvin Sketch software, converted to 3D using MM2 and conformational search was performed using MMFF94 forcefield^57^. The lowest energy conformer was further optimized using MOPAC with AM1 parameterization^58, 59^. The optimized ligands were loaded to MGL Tools one by one to re-assign Gasteiger partial charges and non-polar hydrogen atoms. All torsions (rotatable bonds) were auto detected for ligands and assigned to generate different poses during docking procedure.

Docking of EGFR-TK inhibitors was parameterized with flexible residues to locate the optimum binding conformations of 7a-7o in EGFR-TK protein. Each docking calculation was iterated 100000 times with 250 GA runs, resulting 250 docked poses. For all ligands, random initial positions, random conformers and torsions were used. The translation, quaternion and torsion steps were considered from default values indicated in AutoDock user guide. Lamarckian genetic algorithm was used for minimization using optimum parameters to generate all possible energies to rank the conformers. The parameters for the grid generation and genetic algorithm based docking calculations are shown in Table S2 that is given in supplementary information. The docking parameterization was validated and optimized based on the docking analysis on the active conformation of co-crystal ligand AQ4 - [6,7-Bis(2-Methoxy-Ethoxy)Quinazoline-4-Yl]-(3-Ethynylphenyl)Amine named as Erlotinib (ERL). Re-docking with Autodock Vina was performed for more accuracy based on global optimization and better search algorithm. Best hits were identified and the determination of binding energy to assess the binding affinity of ligands was calculated by employing highest stable ligand-receptor complex through MGL Tools to extract the energy data.

### Statistical analysis

All the data were expressed as the mean ± SEM and analysis of variance (ANOVA) was used for the statistical analysis using Graph Pad Prism version 5.0. The values were considered to be significant when the P value was p < 0.05.

## Electronic supplementary material

Supplementry Information
