# Supplementary material for: Design and discovery of novel monastrol-1,3,5-triazines as potent anti-breast cancer agent via attenuating Epidermal Growth Factor Receptor tyrosine kinase
Source: Sci Rep. 2017 Jul 19;7:5851. doi: 10.1038/s41598-017-05934-5 (PMC5517562; doi:10.1038/s41598-017-05934-5)

Design and discovery of novel monastrol-1,3,5-triazines as potent anti-breast cancer agent *via* attenuating Epidermal Growth Factor Receptor tyrosine kinase

Jitendra Kumar Shrivastava,[1] Girinath G. Pillai[2,3], Hans Raj Bhat,[4] Amita Verma, [1] Udaya Pratap Singh[1]*

[1] *Drug Design & Discovery Laboratory, Department of Pharmaceutical Sciences, Sam Higginbottom University of Agriculture, Technology & Sciences, Allahabad, Uttar Pradesh, India 211007*

[2] *Institute of Chemistry, University of Tartu, Ravila 14a, Estonia 50411*

[3] *Florida Center for Heterocyclic Compounds, University of Florida, Gainesville, FL. 32611*

[4] *Department of Pharmaceutical Sciences, Dibrugarh University, Dibrugarh, Assam 786004*

Supplementary information

Docking Study

Table S1: Protein – Ligand Interaction details.

Ligands	2D Protein-Ligand Interactions	Hydrogen Bond Distances and AA Residues
7a	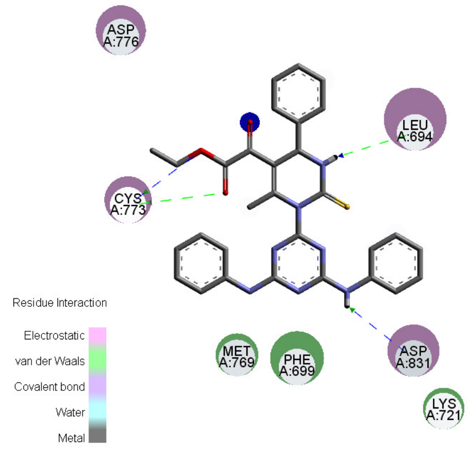	ASP831-H=1.76A^{o}<br>CYS773-H=1.74 & 2.01 A^{o}<br>LEU694-H=2.04A^{o}

7b	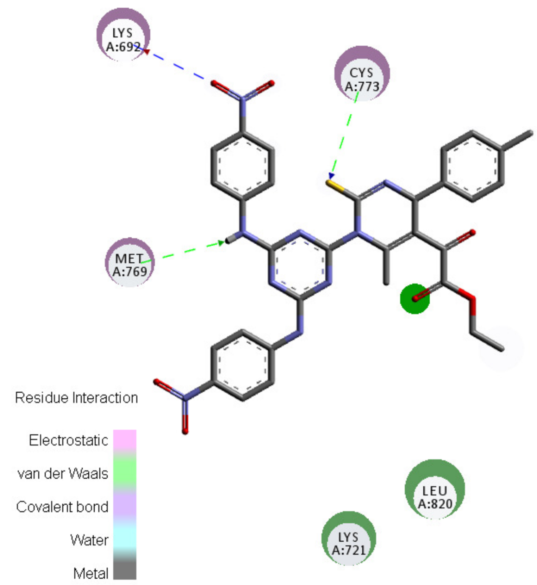	CYS773-H=2.75A^o MET769-H=2.05A^o LYS721-H=2.17A^o LYS692-H=1.73A^o
7c	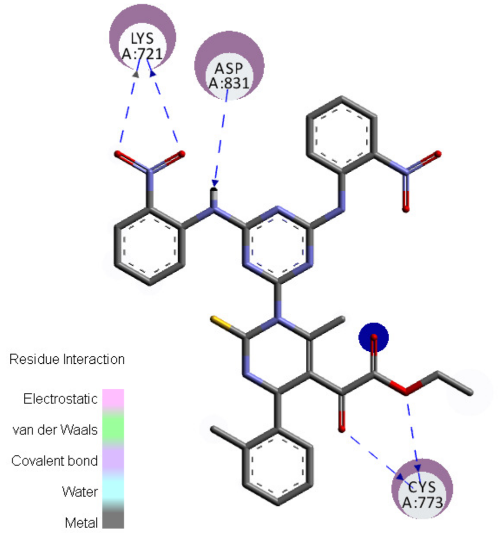	ASP831-H=2.01A^o CYS773-H= 1.80A^o LYS721-H=1.71A^o
7d	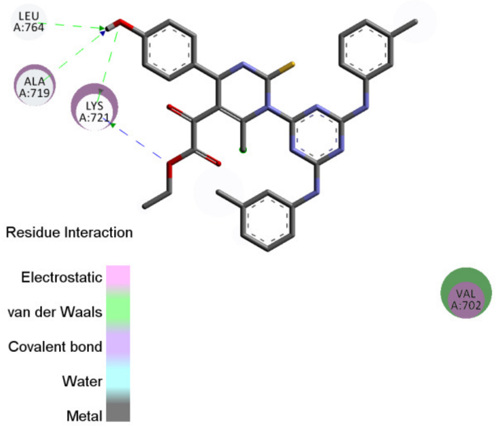	LYS721-H=2.0 A^o

7e	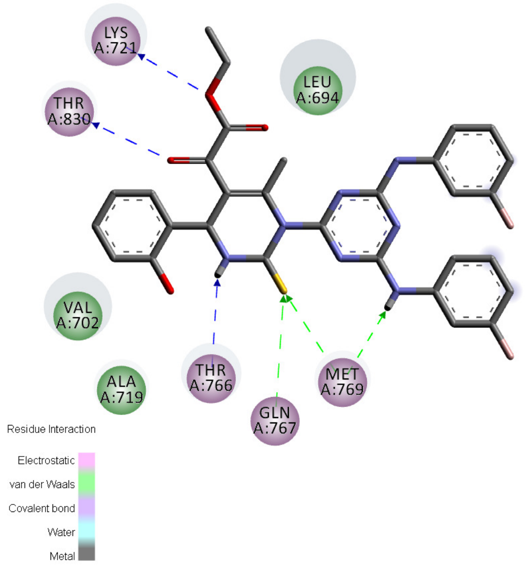	MET769-H=1.83A° LYS721-H=1.66A° GLN767-H=2.84A° THR766-H=2.20A° THR830-H=2.48A°
7f	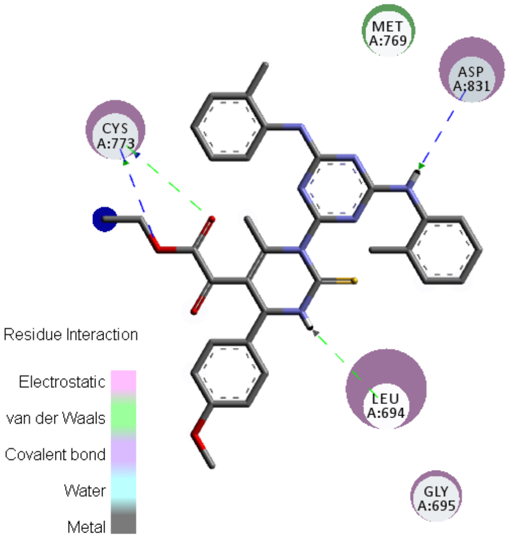	ASP831-H=1.84A° CYS773-H=1.75 & 2.17A° LEU694-H=2.03A°

7g	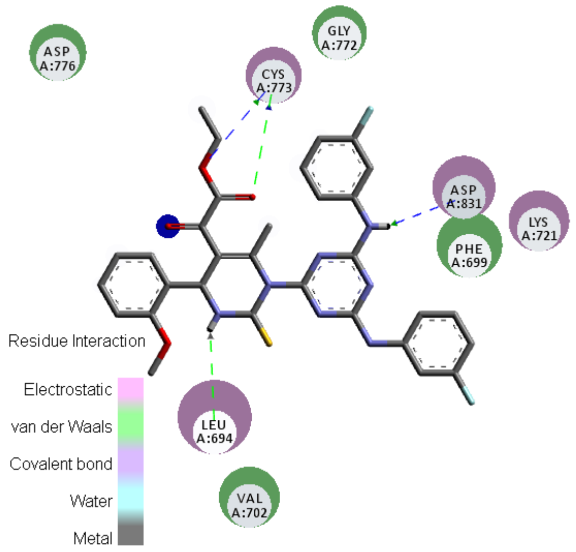	ASP831-H=1.85 A^{o}<br>CYS773-H=1.79 &1.94 A^{o}<br>LEU694-H=2.12 A^{o}
7h	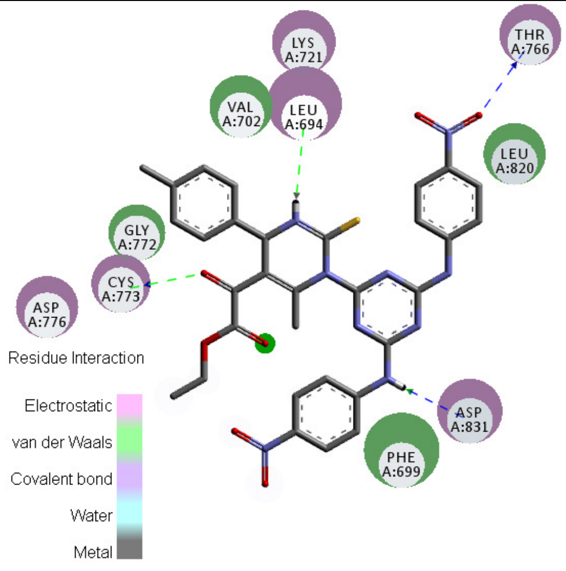	ASP831-H=1.82 A^{o}<br>CYS773-H=2.11 A^{o}<br>LEU694-H=2.34 A^{o}<br>THR766-H=2.04 A^{o}

7i	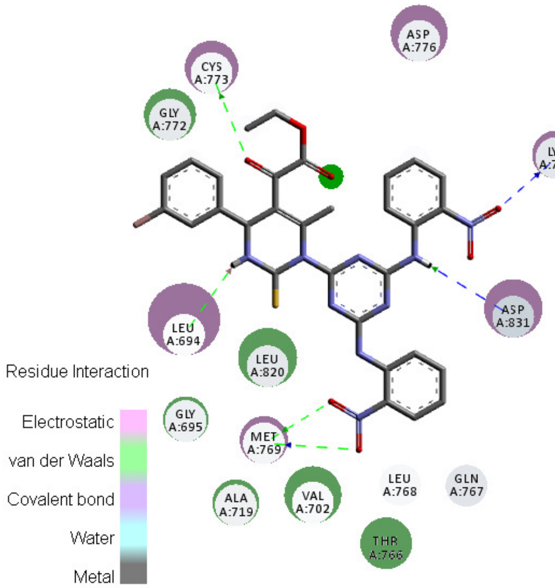 	ASP831-H=2.18 A°<br>CYS773-H=1.90 A°<br>MET769-H=1.96 A°<br>LYS721-H=2.25 A°<br>LEU694-H=2.00 A°
7j	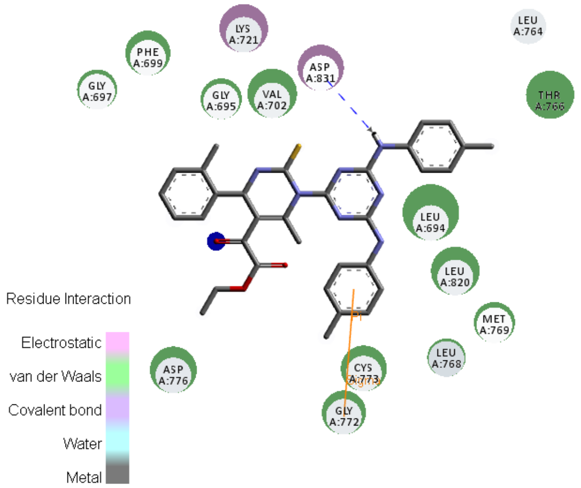 	ASP831-H=2.18A°<br>CYS773-H=1.73 & 2.17A°

7k	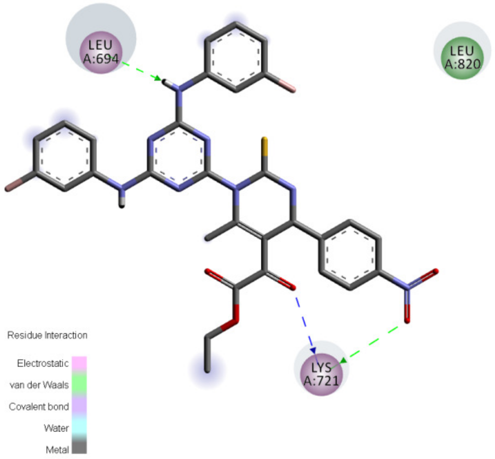	LYS721-H=2.20 &2.41 A^{o} LEU694-H=2.23 A^{o}

7l	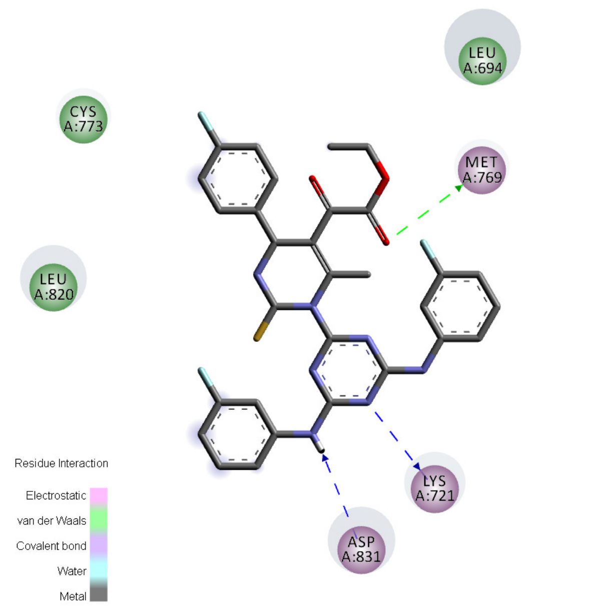	ASP831-H=2.09 A° MET769-H=2.11 A° LYS721-H=1.99 A°
7m	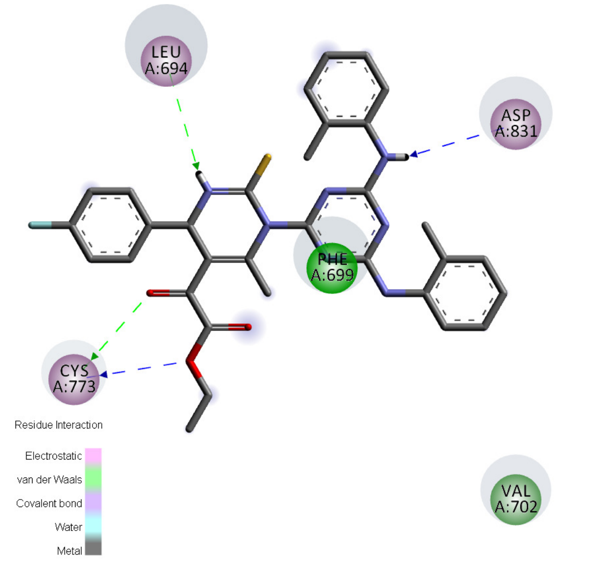	ASP831-H=1.92A° CYS773-H=1.64 A° LEU694-H=2.35 A°

7n	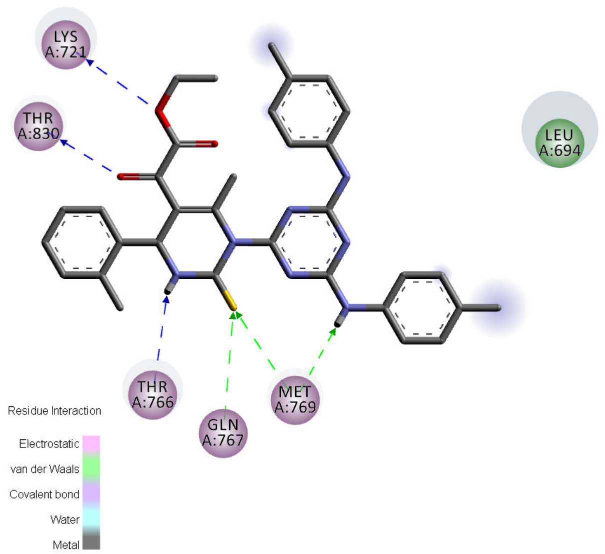	MET769-H=1.96 A^{o} LYS721-H=2.11 A^{o} GLN767-H=2.75 A^{o} THR766-H=2.24 A^{o} THR830-H=2.45 A^{o}
7o	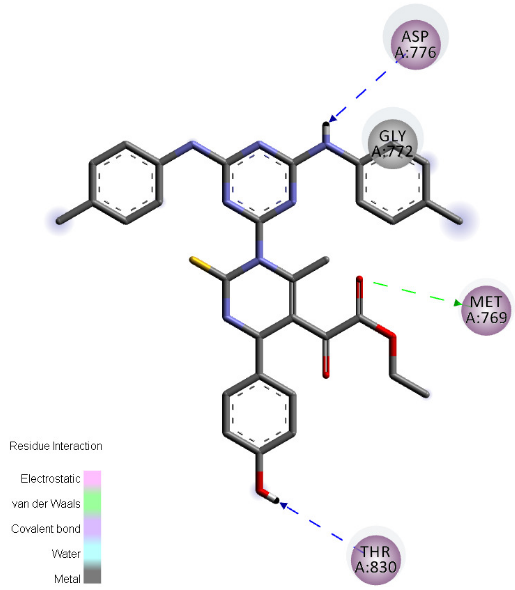	ASP776-H=1.89 A^{o} MET769-H=2.00 A^{o} THR830-H=1.89 A^{o}

<table>
<tr><td>ERL</td><td>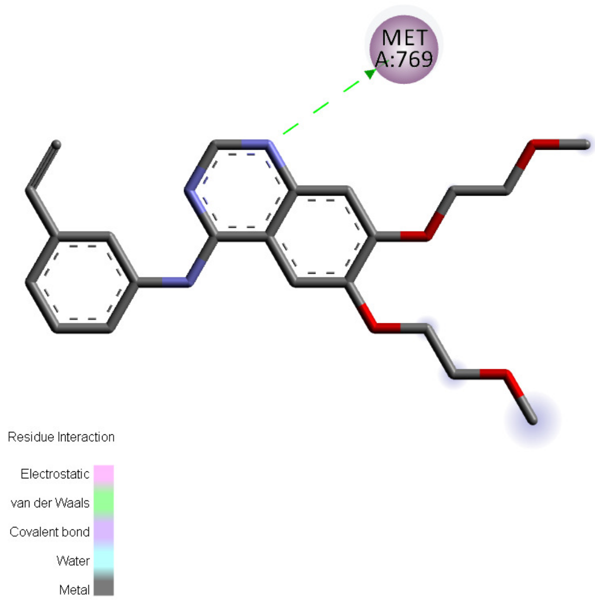
</td><td>MET769-H=1.79 A°</td></tr>
<tr><td>Elements</td><td colspan="2">Description of 2D depiction</td></tr>
<tr><td>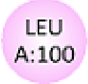
</td><td colspan="2">Residues involved in hydrogen-bond, charge or polar interactions are represented by pink circles.</td></tr>
<tr><td>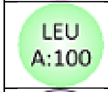
</td><td colspan="2">Residues involved in van der Waals interactions are represented by green circles.</td></tr>
<tr><td>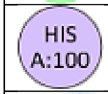
</td><td colspan="2">Covalently bonded residues are represented by magenta-colored circles.</td></tr>
<tr><td>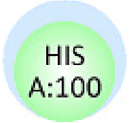
</td><td colspan="2">The solvent accessible surface of an interacting residue is represented by a blue halo around the residue. The diameter of the circle is proportional to the solvent accessible surface.</td></tr>
<tr><td>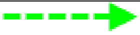</td><td colspan="2">Hydrogen-bond interactions with amino acid main chains are represented by a green dashed arrow directed towards the electron donor.</td></tr>
<tr><td>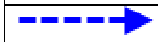</td><td colspan="2">Hydrogen-bond interactions with amino acid side-chains are represented by a blue dashed arrow directed towards the electron donor.</td></tr>
</table>

Table S2: Parameters used in grid generation and docking calculations for Autogrid and Autodock programs respectively.

Grid Parameters		Docking Parameters	
Spacing	0.381Å	Energy evaluations	2500000
	21.715X Å	Iterations	100000
Grid Center	0.349Y Å	Mutation rate	0.02
	52.004Z Å	Crossover rate	0.80
NPTS	42, 40, 40	Elitism value	1
Receptor Type	A, C, H, HD, N, NA, OA, SA	RMS Tolerance	
Ligand Type	A, C, NA, OA, N, HD, F, Br, Cl, SA		

Spectral Data of compound 7a

^{1}H NMR Spectra

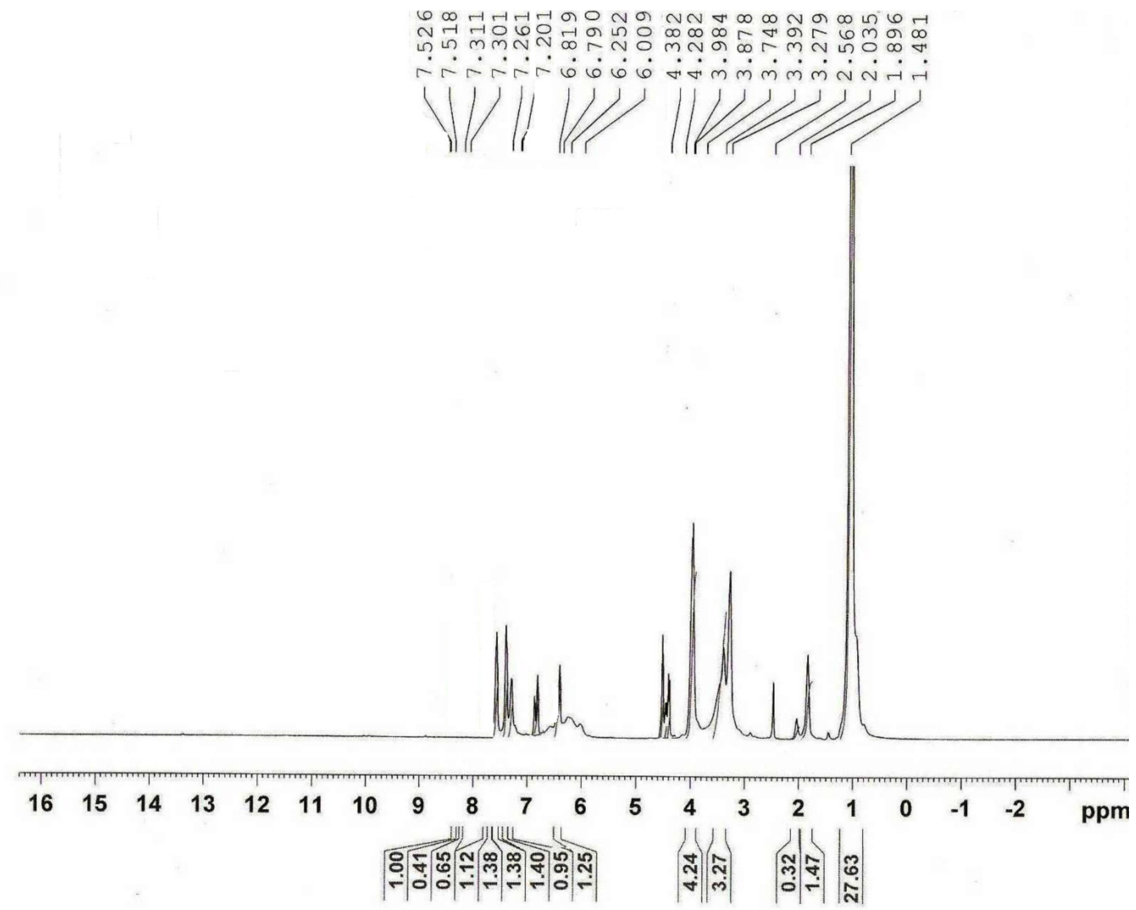

Mass Spectra

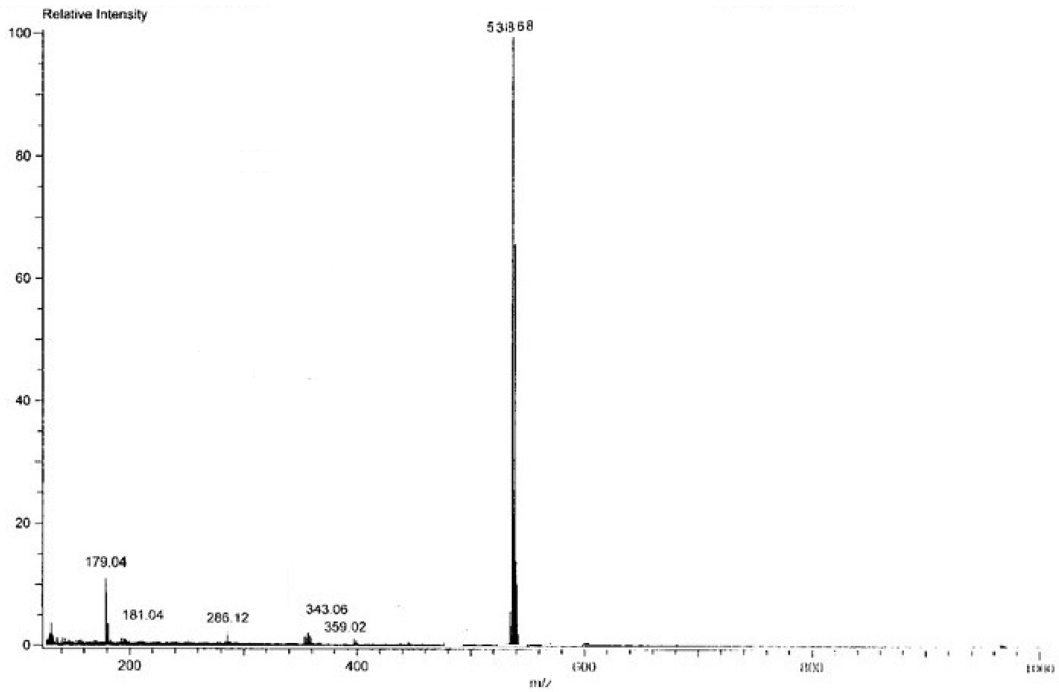

FT-IR Spectra

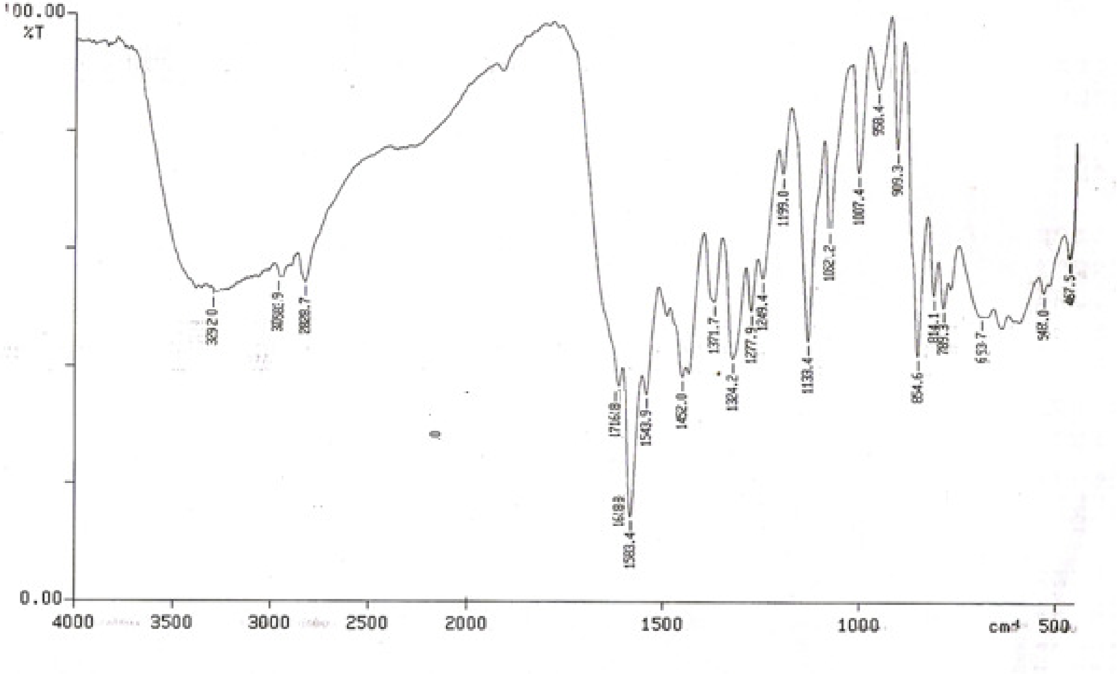

^{13}C- NMR Spectra

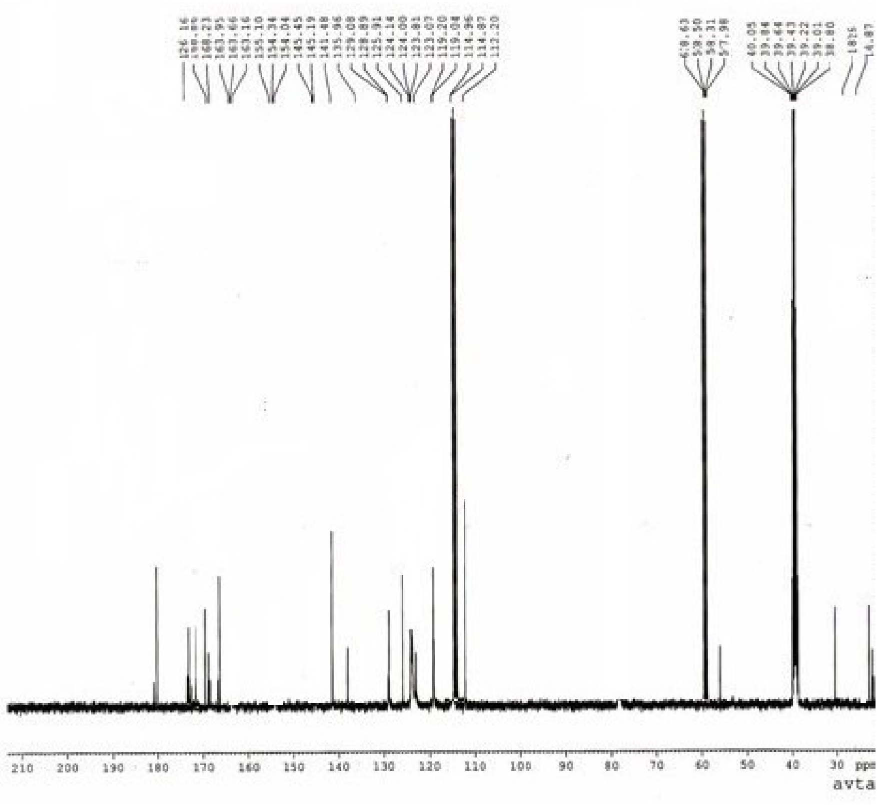

Supplement: Supplementary file 1 — Supplementry Information [file 41598_2017_5934_MOESM1_ESM.pdf]
